# Cancer-prone Phenotypes and Gene Expression Heterogeneity at Single-cell Resolution in Cigarette-smoking Lungs

**DOI:** 10.1158/2767-9764.CRC-23-0195

**Published:** 2023-11-10

**Authors:** Jun Nakayama, Yusuke Yamamoto

**Affiliations:** 1 Laboratory of Integrative Oncology, National Cancer Center Research Institute, Tokyo, Japan.; 2 Department of Oncogenesis and Growth Regulation, Research Institute, Osaka International Cancer Institute, Osaka, Japan.

## Abstract

Single-cell RNA sequencing (scRNA-seq) technologies have been broadly utilized to reveal molecular mechanisms of respiratory pathology and physiology at single-cell resolution. Here, we established single-cell meta-analysis (scMeta-analysis) by integrating data from eight public datasets, including 104 lung scRNA-seq samples with clinicopathologic information and designated a cigarette-smoking lung atlas. The atlas revealed early carcinogenesis events and defined the alterations of single-cell transcriptomics, cell population, and fundamental properties of biological pathways induced by smoking. In addition, we developed two novel scMeta-analysis methods: VARIED (Visualized Algorithms of Relationships In Expressional Diversity) and AGED (Aging-related Gene Expressional Differences). VARIED analysis revealed expressional diversity associated with smoking carcinogenesis. AGED analysis revealed differences in gene expression related to both aging and smoking status. The scMeta-analysis paves the way to utilize publicly-available scRNA-seq data and provide new insights into the effects of smoking and into cellular diversity in human lungs, at single-cell resolution.

**Significance:**

The atlas revealed early carcinogenesis events and defined the alterations of single-cell transcriptomics, cell population, and fundamental properties of biological pathways induced by smoking.

## Introduction

Smoking is the leading risk factor for early death, and its negative effects present individual and public health hazards (1, 2). Cigarette smoke is a mixture of thousands of chemical compounds generated from tobacco burning (3) that causes chronic airway inflammation, reactive oxygen species (ROS) production, and DNA damage. Specifically, it has been discovered that smoking injures the respiratory organs and cardiovascular system and causes carcinogenesis, chronic obstructive pulmonary disease, and atherosclerosis (4). In particular, the incidence of lung squamous carcinoma (LUSC) is significantly increased by cigarette smoking (5, 6).

Single-cell RNA sequencing (scRNA-seq) technologies have been broadly utilized to reveal the molecular mechanisms of respiratory diseases and physiology at single-cell resolution. scRNA-seq in human lungs identified novel cell populations and cellular diversity (7–13). However, there are several concerns regarding scRNA-seq analysis. One of these concerns is sample size, that is, that clinical scRNA-seq analyses could be biased because of insufficient sample sizes. A possible solution is meta-analysis of scRNA-seq data. The recently developed single-cell meta-analysis (scMeta-analysis) method has been considered a powerful tool for large-scale analysis of integrated single-cell cohorts. The scMeta-analysis shows robust statistical significance and the capacity to compare the results among different studies at the single-cell level. In fact, integrated scMeta-analysis of a number of cohorts has revealed a previously unappreciated diversity of cell types and gene expression; for example, scMeta-analysis of lung endothelial cells, including human and mouse datasets, revealed novel endothelial cell populations (14–17). In addition, comparative analysis of scRNA-seq cohorts revealed pan-cancer tumor-specific myeloid lineages (18).

In this study, we integrated eight publicly available datasets comprising 104 lung scRNA-seq samples and analyzed a total of 230,890 single cells to construct a cigarette-smoking lung atlas. The scMeta-analysis of the cigarette-smoking lung atlas defined single-cell gene expression according to smoking, age, and gender. In addition, we developed novel scMeta-analysis methods: VARIED (Visualized Algorithms of Relationships In Expressional Diversity) analysis and AGED (Aging-related Gene Expressional Differences) analysis with clinical metadata. VARIED analysis revealed the diversity of gene expression associated with cancer-related events in each cell population, and AGED analysis revealed the expressional differences in relation to both aging and smoking status.

## Materials and Methods

### scRNA-seq Data Collection from Public Databases

The scRNA-seq cohorts were downloaded from the public Gene Expression Omnibus (GEO) and European Genome-Phenome Archive (EGA) databases (Supplementary Table S1). We collected scRNA-seq samples of human lungs for which smoking status information was available. From physiologic studies of the lung airway, all 10 never-smoker samples were extracted from the EGA00001004082 dataset (19), and one never-smoker and three smoker samples were extracted from the GSE130148 dataset (13). From idiopathic pulmonary fibrosis studies, five never-smoker and three smoker samples were extracted from a total of 17 samples in the GSE122960 dataset (20), one never-smoker and seven smoker samples were extracted from a total of 34 samples in the GSE135893 dataset (12), and 22 never-smoker and 23 smoker samples were extracted from a total of 78 samples in the GSE136831 dataset (11). From studies of lung disease in smokers, three never-smoker and three smoker samples were extracted from the GSE123405 dataset (21), and three never-smoker and nine smoker samples were extracted from the GSE173896 dataset (22). From lung cancer studies, four never-smoker and seven smoker samples were extracted from a total of 58 samples in the GSE131907 dataset (23). A total of 104 samples (never-smoker: 49, smoker: 56) were collected, and the details of the extracted samples are shown in Supplementary Table S2. These datasets were imported into R software version 4.2.0. (RRID:SCR_001905) and transformed into Seurat objects with the package Seurat version 4.3.0 (ref. 24; RRID:SCR_016341). The Seurat objects from the different datasets were then integrated in R.

### Integration of Datasets, Data Quality Control, and Removal of Batch Effects

The integrated atlas of eight pubicly datasets was subjected to normalization, scaling, and principal component analysis with Seurat functions. Removal of low-quality cells was performed against the merged dataset before batch effect removal according to the following criteria (nFeature_RNA > 1,000 and percent.mt < 20). The expression counts of each sample were normalized by “SCTransform” method version 0.3.5. (RRID:SCR_022146; ref. 25). Doublet cells in the integrated atlas were removed by “DoubletFinder” method version 2.0.3. (refs. 26, 27; RRID:SCR_018771). To remove the batch effect between cohort studies, Harmony version 0.1.1. algorithms (RRID:SCR_022206) were applied to the integrated atlas (28, 29) following the instructions in the Quick start vignettes (https://portals.broadinstitute.org/harmony/articles/quickstart.html). cLISI score and iLISI score were calculated by “lisi” package (28) using cell annotation metadata or dataset metadata.

### Cell Type Annotation and Cell-cycle Scoring

Clustering of neighboring cells was performed by the functions “FindNeighbors” and “FindClusters” from Seurat using Harmony reduction. First, the clusters were grouped on the basis of the expression of tissue compartment markers (e.g., *EPCAM* for epithelia, *CLDN5* for endothelia, *COL1A2* for fibroblasts, and *PTPRC* for immune cells) and then annotated in detail according to “A molecular cell atlas of the human lung” (7). Cell-cycle analysis was performed with the “CellCycleScoring” function of Seurat.

### VARIED Analysis

To evaluate the expressional heterogeneity in the cell populations, we calculated the correlation coefficients for each cell population between smokers and never-smokers. In each cluster, normalized closeness centrality was calculated in R using ggraph version 2.1.0. (RRID:SCR_021239) and igraph version 1.3.5. packages (RRID:SCR_019225), as described previously (22, 30, 31). We calculated the differences of median of normalized closeness centrality between smokers and never-smokers in each cluster. Extraction of differentially expressional genes (DEG) between smoker basal-differentiated (basal-d) cells and never-smoker basal-d cells was performed by “FindAllMarkers” functions in Seurat where *r* is the absolute value of Pearson correlational coefficient and *n* is the number of cells in the cluster.



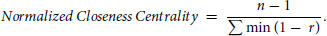



### Gene Set Variation Analysis Scoring and Survival Analysis

Gene set variation analysis (GSVA) was performed using RNA-seq dataset of The Cancer Genome Atlas (TCGA) LUSC cohorts by “GSVA” package version 1.44.5. (RRID:SCR_021058) in R (32). RNA-seq dataset and clinical information of the patients with LUSC were downloaded from TCGA Data Portal (ref. 33; RRID:SCR_014514). The signature genes of basal-d smoker clusters showed in Supplementary Tables S3 and S4. Scoring method by “gsva” algorithms was utilized for calculation of enrichment score in the patients with lung squamous cancer. We subjected clinical status and gene expression data to survival analysis using “survminer” version 0.4.9. (RRID:SCR_021094) and “survival” packages version 3.4.0 (RRID:SCR_021137) in R. TCGA dataset is available at https://portal.gdc.cancer.gov/.

### Module Analysis

Module analysis was performed by the “AddModuleScore” function in Seurat using the gene lists from MSigDB (https://www.gsea-msigdb.org/gsea/msigdb/, RRID:SCR_016863). The epithelial-to-mesenchymal transition (EMT) module (HALLMARK_EPITHELIAL_MESENCHYMAL_TRANSITION), heme metabolism module (HALLMARK_HEME_METABOLISM), ROS module (HOUSTIS_ROS), autophagy module (REACTOME_AUTOPHAGY), IFN signaling module (REACTOME_INTERFERON_SIGNALING), senescence module (REACTOME_CELLULAR_SENESCENCE), circadian module (REACTOME_CIRCADIAN_CLOCK), mitophagy module (REACTOME_MITOPHAGY), pyroptosis module (REACTOME_PYROPTOSIS), and ferroptosis module (WP_FERROPTOSIS) were subjected to module analysis in each cell population.

### Pathway Enrichment Analyses and Ingenuity Pathway Analysis

We performed enrichment analysis against the marker gene list in each cluster between male and female smokers by the “ClusterProfiler” version 4.4.4. (RRID:SCR_016884; ref. 34) and “ReactomePA” version 1.40.10. (RRID:SCR_019316; ref. 35) packages in R. Gene symbols were converted to ENTREZ IDs using the “org.Hs.eg.db” package version 3.10.0. Pathway datasets were downloaded from the Reactome database (RRID:SCR_003485). Pathway enrichment analysis using the “enrichPathway” function was performed by the Benjamini-Hochberg (BH) method. Marker genes of the basal-d cluster in smokers and never-smokers were calculated by “FindMarkers” with the MAST method (RRID:SCR_016340; ref. 36). Enrichment analysis of basal-d was performed using QIAGEN Ingenuity Pathway Analysis (IPA) software (RRID:SCR_008653).

### AGED Analysis

We calculated the average expression of all cells in each cluster in both smokers and never-smokers and performed regression analysis in correlation with gene expression and patient age by R. Next, we calculated the differences in slopes (delta) in smokers and never-smokers via regression analysis. We extracted the genes with the differences in slopes (Δ) value over 0.5 in three or more types of cell cluster. Extracted genes were shown in a heat map.

### Data Visualization

The dimensionality-reduced cell clustering is shown as a Uniform Manifold Approximation and Projection (UMAP) plot by the function “runUMAP”. Heat maps were drawn by Morpheus from the Broad Institute (RRID:SCR_014975). A ridge plot was drawn using the “ggridges” version 0.5.4. package (RRID:SCR_024511) in R. Violin plots were drawn using the “ggplot2” version 3.4.0. package (RRID:SCR_014601) in R.

### Statistical Analysis

Correlation coefficients were calculated by Spearman correlation in R. Welch *t* test or Tukey or Dunnett multiple comparison test was used for comparison of the datasets. log-rank test was used for survival analysis in R. Significance was defined as *P* < 0.05.

### Data Availability Statement

scMeta-analysis data were available to the NCBI GEO database and EGA database. Detailed information is shown in Supplementary Table S1. The datasets GSE122960, GSE123405, GSE130148, GSE131907, GSE135893, GSE136831, and GSE173896 are available in the NCBI GEO database (https://www.ncbi.nlm.nih.gov/geo/). The EGAS00001004082 dataset is available in the EGA database (https://ega-archive.org/). The source code of scMeta-analysis is available on GitHub (https://github.com/JunNakayama/scMeta-analysis-of-cigarette-smoking). The integrated atlas is available in FigShare (https://figshare.com/projects/Single-cell_meta-analysis_of_cigarette_smoking_lung_atlas/175497). The rest of the data in the study are available from the corresponding authors.

## Results

### Establishment of Integrated Single-cell Lung Atlas with Cigarette Smoking

**FIGURE 1:**
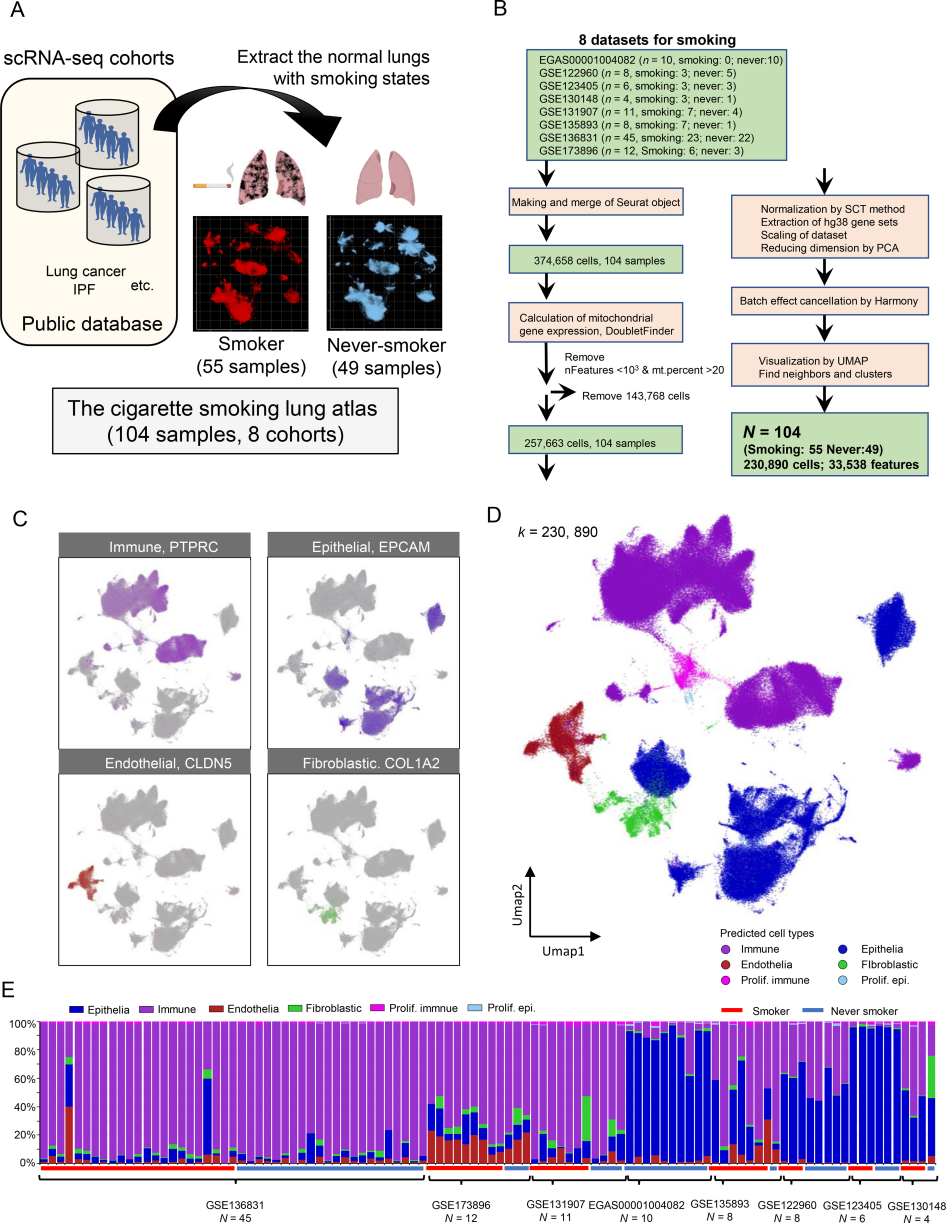
Establishment of the integrated lung atlas with cigarette smoking status from eight scRNA-seq cohorts. **A,** Overview of the establishment of the integrated lung atlas with cigarette smoking status. The control lung scRNA-seq data from eight publicly available datasets were obtained and integrated with smoking status information. **B,** Flow diagram of the establishment of the integrated scRNA-seq lung atlas with cigarette smoking. Eight publicly available scRNA-seq datasets were downloaded and combined in Seurat. Doublet cells were removed by DoubletFinder. The datasets were normalized by SCTramsform, integrated by Harmony to adjust for batch effects. **C,** Representative marker expression patterns for the cell type clusters shown in the UMAP plot. **D,** A UMAP plot displaying 230,890 single human lung cells of 55 smokers and 49 never-smokers. Each dot represents a single cell, and cell clusters are classified as immune cells, epithelial cells, endothelial cells, and fibroblasts. **E,** Cell populations of immune cell, epithelial cell, endothelial cell, and fibroblast clusters across the 104 samples in the integrated atlas. Smokers, 55 cases; never-smokers, 49 cases.

According to scRNA-seq collection criteria, we chose eight publicly available datasets of lung scRNA-seq data to construct a cigarette-smoking lung atlas (Fig. 1A). To this end, we collected data from 374,658 single cells from 104 scRNA-seq samples (smoker: 55 samples, never-smoker: 49 samples; Fig. 1A). Integrated single-cell transcriptome data were linked with clinical metadata such as smoking status, age, gender, and race (Supplementary Tables S1 and S2; Supplementary Fig. S1A). In the process of quality control with Seurat in R, 143,768 low-quality single cells (nFeatures < 10^3^ and mt.percent > 20%) were removed. Doublet cells were computationally removed by “DoubletFinder” algorithm (26). The single cells were normalized by SCTransform method in Seurat (25). Integration of the eight datasets was performed by the Harmony algorithm with the smoking status of scRNA-seq samples (ref. 28; Fig. 1B). To confirm that the integration of the eight datasets reduced bias, we showed the atlas marked with the datasets (Supplementary Fig. S1B). Median of cLISI scores is 1.00, median of iLISI score is 2.21, suggesting that the datasets are well mixed by Harmony (Supplementary Fig. S1C). The normalized dataset was integrated for removal of batch effect by Harmony algorisms (Supplementary Fig. S1B). The cigarette-smoking lung atlas is composed of a total of 230,890 single cells. The density plot showed that the majority of single cells in the atlas were immune cells and epithelial cells (Supplementary Fig. S1D). UMAP plots with cell type–specific markers (*PTPRC* as an immune marker, *EPCAM* as an epithelial marker, *CLDN5* as an endothelial marker, and *COL1A2* as a fibroblast marker) showed an obvious segregation of immune, epithelial, endothelial, and fibroblastic lineages (Fig. 1C and D). There were 118,364 single cells in the smoker group and 112,526 single cells in the never-smoker group (Supplementary Fig. S2A). Comparison of the atlases by smoking status revealed that most of the cell populations in the UMAP plot overlapped; however, parts of epithelial clusters were specific to the never-smoker group (Supplementary Fig. S2A). All major clusters seemed to overlap among the eight datasets (Supplementary Fig. S2B), although the populations of cells were different in each dataset (Fig. 1E). This difference in cell populations could be caused by differences in tissue collection and cell isolation processes.

**FIGURE 2:**
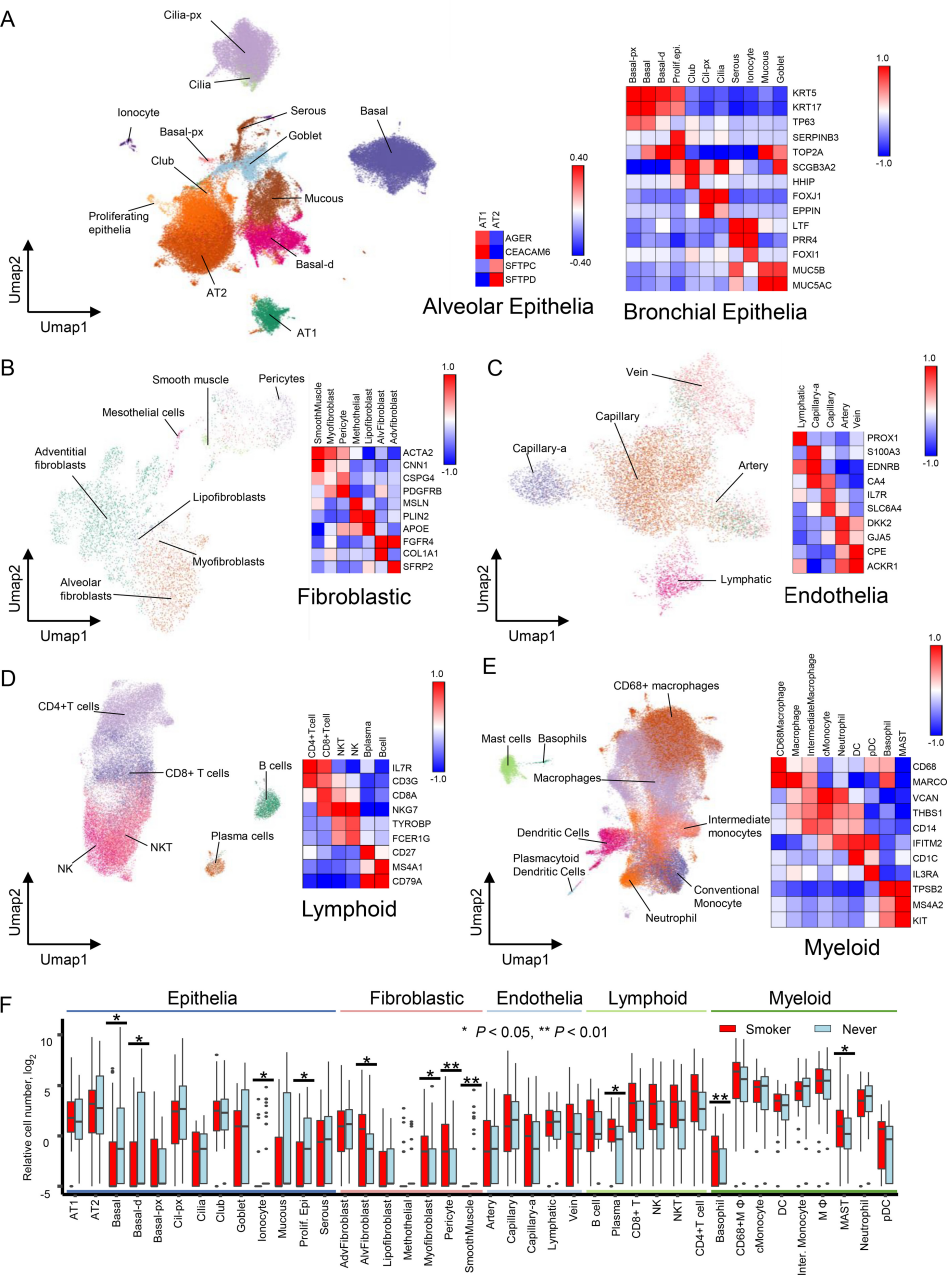
Cell-type classification of the integrated lung atlas. UMAP plots for each cell type cluster. The UMAP plot of the cigarette-smoking lung atlas was divided into five UMAP plots based on the cell-type clusters. Heat maps of selected marker genes in each cell type cluster. Each cluster was defined according to marker expression profiles. **A,** Epithelia: 13 clusters. **B,** Fibroblasts: 7 clusters. **C,** Endothelial cells: 5 clusters. **D,** Lymphoid cells: 6 clusters. **E,** Myeloid cells: nine clusters. **F,** Relative cell number plots between smokers and never-smokers in 39 cell types in the integrated atlas. Welch *t* test; *, *P* < 0.05. Blue line: epithelial cell types, pink line: fibroblastic cell types, light blue line: endothelial cell types, light green line: lymphoid cell types, and green line: myeloid cell types.

For detailed reannotations with all cell types in the integrated atlas, we first identified the cell types present within the atlas according to the lung cell markers in the human lung scRNA-seq atlas (ref. 7; Fig. 2; Supplementary Fig. S3). To investigate the cell types in further detail, we extracted subsets of “epithelia” (Fig. 2A), “fibroblasts” (Fig. 2B), “endothelia” (Fig. 2C), “lymphoids” (Fig. 2D), and “myeloids” (Fig. 2E) repeated the UMAP procedure with each subset, which comprised 39 subpopulations in total. There were 13 epithelial cell types (smoker: 24,084 cells, never-smoker: 53,754 cells; Supplementary Fig. S4), seven fibroblastic cell types (smoker: 3,081 cells, never-smoker: 1,592 cells; Supplementary Fig. S5), five endothelial cell types (smoker: 7,600 cells, never-smoker: 3,783 cells; Supplementary Fig. S6), six lymphoid cell types (smoker: 25,331 cells, never-smoker: 10,929 cells; Supplementary Fig. S7), and myeloid cell types (smoker: 55,398 cells, never-smoker: 40,824 cells; Supplementary Fig. S8).

Cigarette smoking is known to induce alterations in cell populations in the lungs. For example, the number of basal-linage cells decreased (37), and the number of basophils increased (38) in smoking lungs. The atlas showed differences in the numbers of 39 cell subpopulations by smoking status (Fig. 2F). Evidently, the cell numbers of basal, basal-d, ionocyte, and proliferating epithelia clusters significantly decreased. Previous bulk studies have reported that the number of bronchial epithelial cells is altered by smoking (9, 37, 39). Consistent with these reports, our data confirmed that smoking had a devastating effect on epithelial cells in the bronchus and bronchiole. The integrated atlas confirmed the increase in basophil cell number with smoking. We also examined the cell cycle in each cell cluster. The cell cycle indices in each subpopulation were not obviously changed between the smoking and never-smoking groups (Supplementary Fig. S9).

### VARIED Analysis Visualized Variations in Epithelial Populations and Basophils by Smoking Status

**FIGURE 3:**
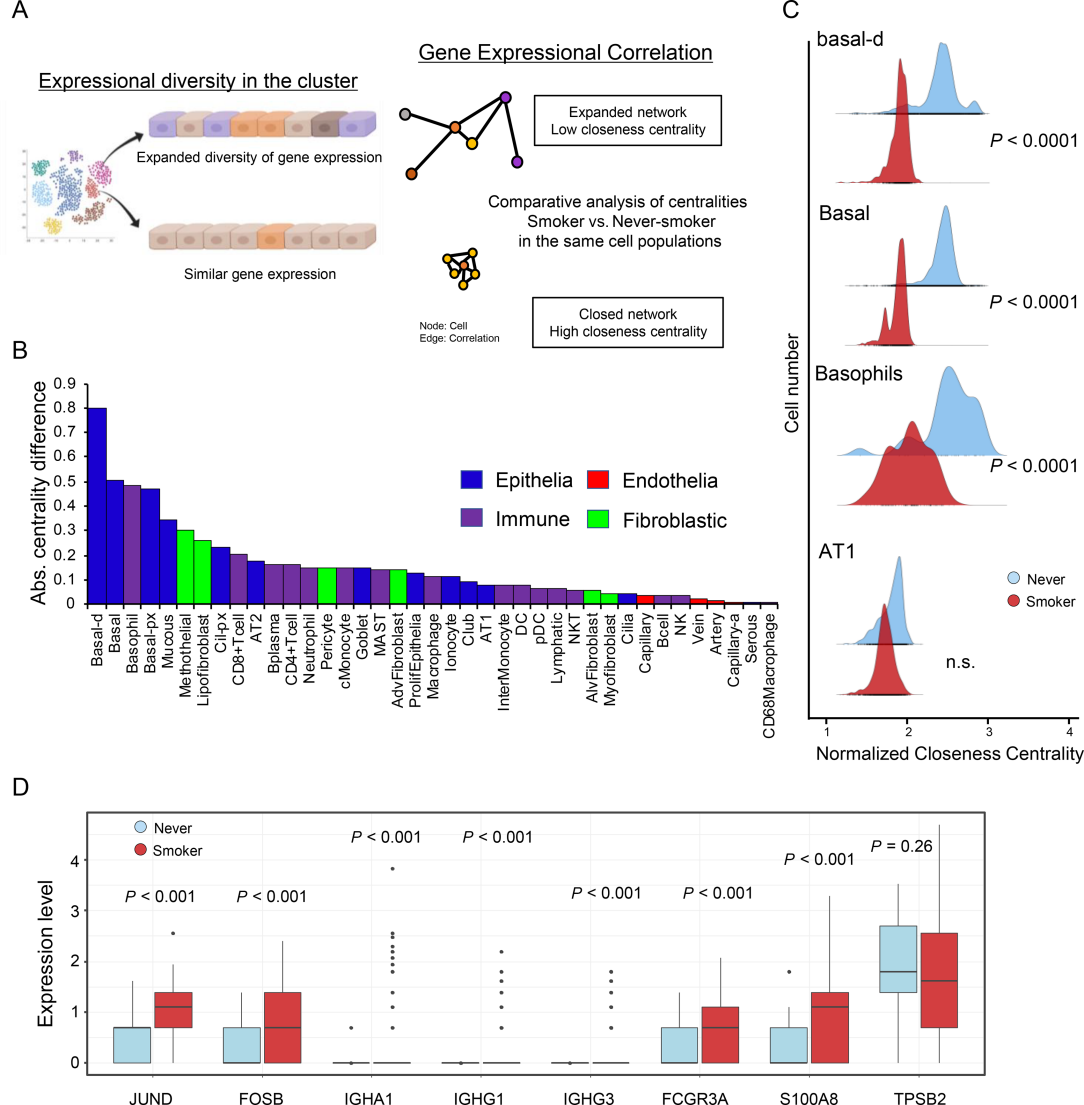
VARIED analysis for cellular variations by smoking status. **A,** Schematic of VARIED analysis for quantifying the alterations in gene expressional diversity between smokers and never-smokers. In each single cell from scRNA-seq, the closeness centrality was calculated in the cell types between smokers and never-smokers. A part of the figure was created with BioRender.com. **B,** Plot of absolute values of difference in centrality in each cell type cluster. Blue: epithelia, purple: immune cells, red: endothelia, and green: fibroblasts. **C,** Representative ridge plots for the closeness centrality between smokers and never-smokers. Welch *t* test. **D,** Expression profiles of activation marker gene in the basophil clusters. Box plots of *JUND*, *FOSB*, *IGHA1*, *IGHG1*, *IGHG3*, *FCGR3A*, *S100A8*, and *TPSB2* between smokers and never-smokers in the basophil cluster. Welch *t* test.

Cigarette smoking is the highest risk factor for carcinogenesis of squamous carcinoma in the bronchia and trachea of the lung (2, 5). To comprehensively understand the effects of smoking in the lung, we developed VARIED analysis to quantify the alteration in gene expressional diversity. VARIED analysis is based on the network centrality of a correlational network with graph theory in each single cell. Previously, we reported that the topology of cell-cell correlational network shows the alteration of cell population (22, 31). In this study, to identify the gene expressional alteration by smoking, we calculated the differences of closeness centrality between smoker and never-smoker. When the random sampling number of the cells is over 100 cells, the medians of VARIED converged to a certain value (Supplementary Fig. S10). Therefore, VARIED analysis needed over 100 cells to calculate the robust centralities. In this study, because all clusters have over 100 cells, we subjected VARIED analysis into the clusters of smoker and never-smoker. The differences in the centrality between smokers and never-smokers represent the alteration of gene expressional diversity in each cell cluster (Fig. 3A). VARIED analysis revealed greater diversity in epithelial clusters, suggesting that cigarette smoking primarily perturbed epithelial populations, particularly in the bronchia and trachea (Fig. 3B and C). These data are consistent with the fact that epithelial cells, located at the bronchia, are considered to be the origin of LUSC (40). Interestingly, the diversity in basophils was also remarkably altered by cigarette smoking. Basophils are known to be activated as a protective immunity against helminths and ticks by expression of cytokines and Immunoglobins (41). Basophils in the smokers expressed *JUND*, *FOSB*, *IGHA1*, *IGHG1*, *IGHG3*, *FCGR3A*, and *S100A8* (Fig. 3D), suggesting their activation and IgG production in smoker lungs.

**FIGURE 4:**
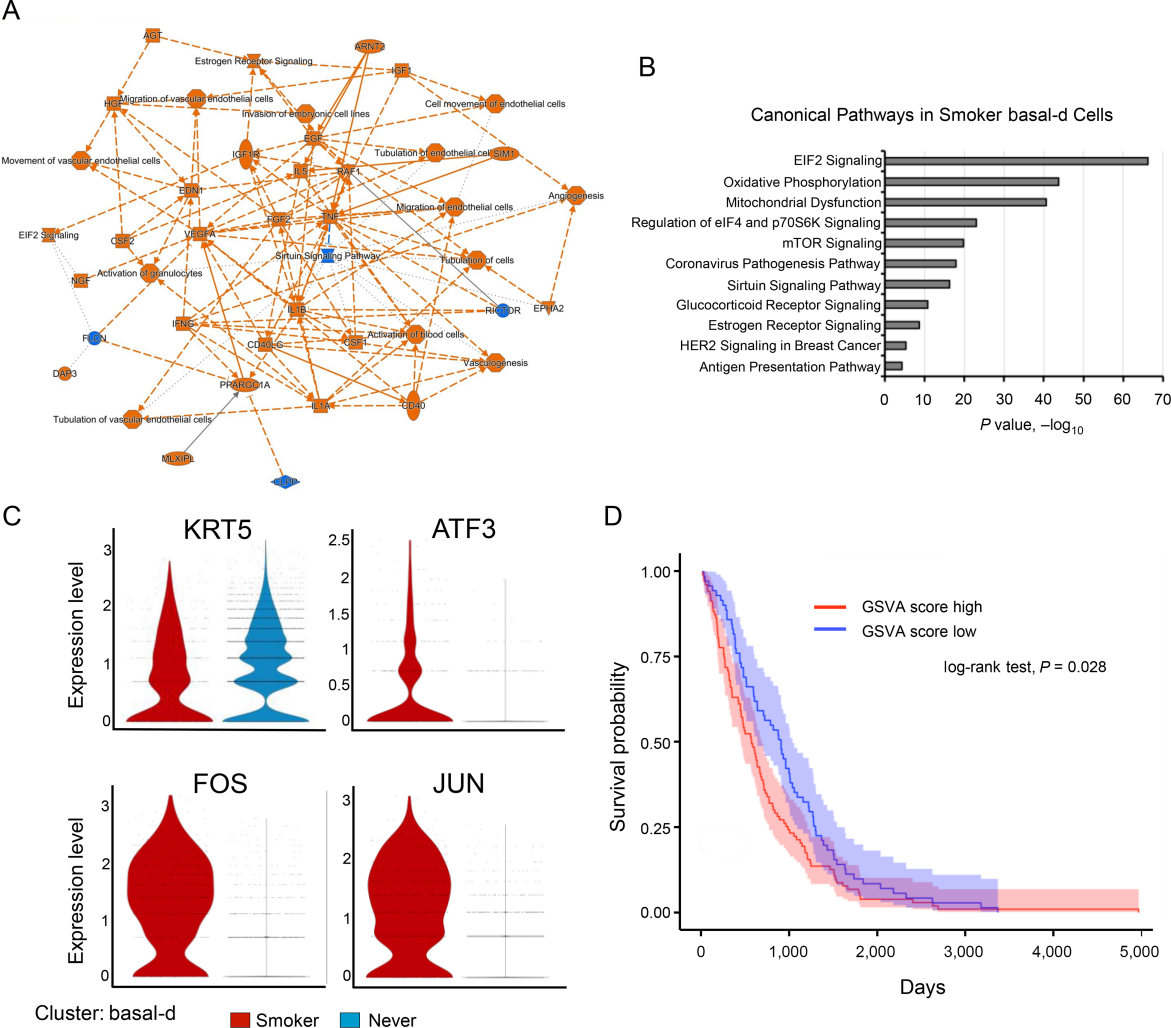
Cancer-related alteration in the basal-d cluster. Expression profiles of DEGs in the basal-d clusters between smokers and never-smokers. **A,** Gene and pathway networks of marker genes for the basal-d cluster. The network plot was generated by IPA. **B,** Enrichment analysis of marker genes for the basal-d cluster. Significantly enriched pathways are shown based on IPA data. **C,** Marker expression patterns in the smoker basal-d clusters. Violin plots of *KRT5*, *ATF3*, *FOS*, and *JUN* between smokers and never-smokers. Welch *t* test **D.** Survival analysis of LUSC cohorts in TCGA with basal-d smoker signature genes. GSVA scores using smoking basal-d signature genes were calculated by GAVA algorithm. log-rank test.

To examine the molecular basis for diversity in gene expression, we extracted differentially expressed genes (DEG) in the basal-d cluster between smokers and never-smokers, focusing on basal-d because this cluster was the most influenced by cigarette smoking (Fig. 4; Supplementary Table S3). Enrichment analysis of the DEGs revealed that protein production–related signal, mitochondrial dysfunction pathways were significantly enriched in the smoker basal-d cluster (Fig. 4A and B; Supplementary Table S5). Our data indicate that smoking adversely affects bronchial epithelial cells and alters gene expressional diversity in carcinogenesis. The basal-d cluster in smoker significantly highly expressed *ATF3*, *FOS*, and *JUN* (Fig. 4C). The VARIED analysis suggested the early oncogenic events in bronchial and tracheal epithelial cells. In addition, the GSVA score using the signature genes of smoker basal-d cluster correlated to poor prognosis in LUSC cohorts in TCGA (Fig. 4D; Supplementary Table S5).

### Cigarette smoking affected genome-wide association study–related genes in LUSC

As the cigarette-smoking lung atlas provided high-resolution expression data in 39 cell types, we explored gene expression profiles from a genome-wide association study (GWAS) of LUSC with smoking (42). To identify the expressional patterns and the broad contributions of different lung cell types to squamous carcinoma susceptibility, the expression levels of an average of 92 GWAS genes were examined in all lung cell types (Supplementary Fig. S11A). High expression of squamous carcinoma GWAS genes was observed in the specific clusters, and cigarette smoking affected the expression of GWAS-related genes in some clusters. In particular, the expression of *MUC1* was increased in the smoker epithelial clusters, and the expression of *HLA-A* was increased in the smoker myeloid clusters (Supplementary Fig. S11B). Mutated *MUC1* has oncogenic roles in carcinogenesis in the human lung (43, 44). Truncating mutations in *HLA-A* carry a risk of dysregulation of cancer-related pathways (45).

### Cancer-associated alterations induced by smoking

**FIGURE 5:**
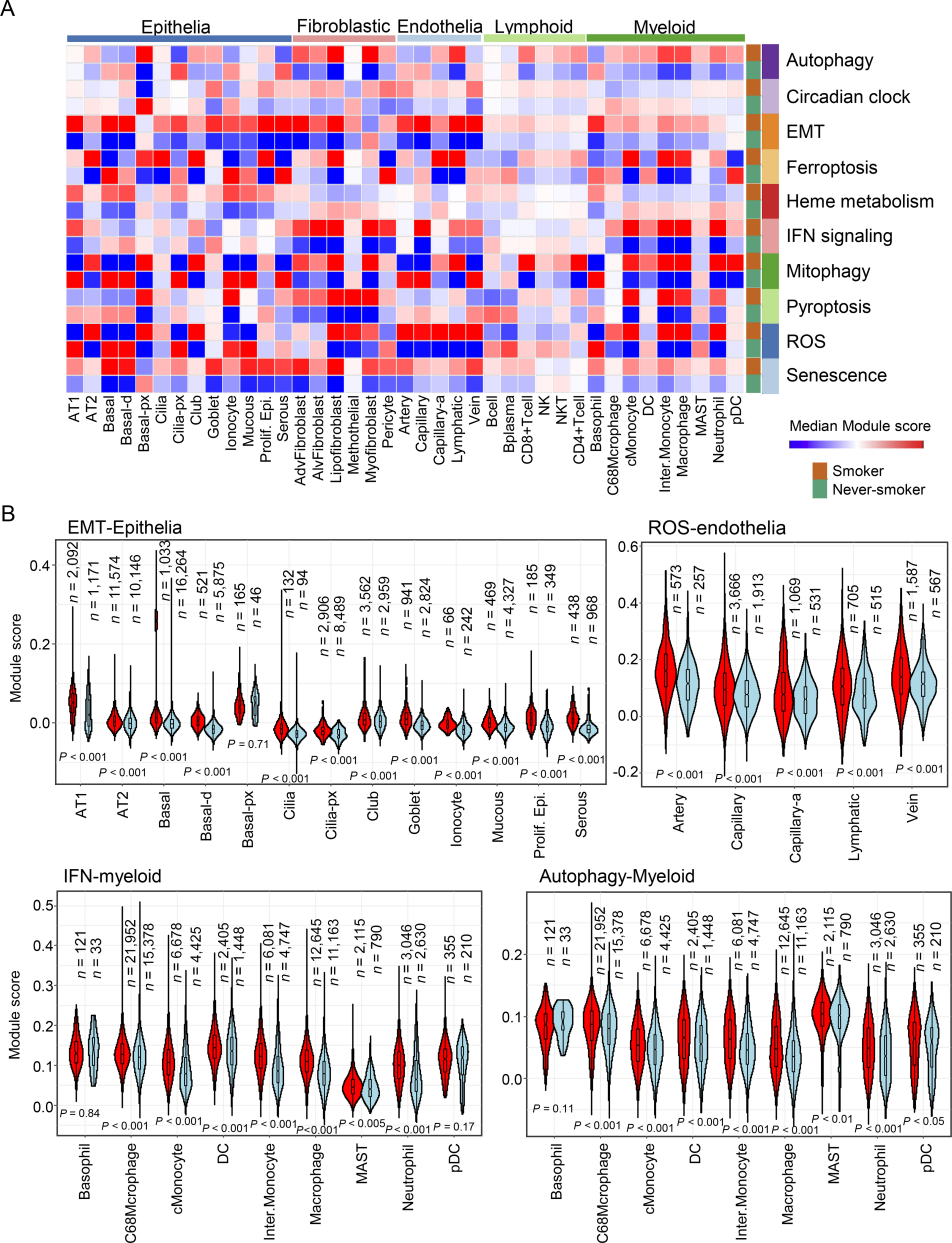
Module analysis of cancer-related pathways in all cell types between smokers and never-smokers. **A,** A heat map of module analysis between smokers and never-smokers across the cell types. The median module score was calculated for each cell type. The senescence, ROS, pyroptosis, mitophagy, IFN signaling, heme metabolism, ferroptosis, EMT circadian clock, and autophagy modules are shown in the heat map. **B,** Violin plots for analysis of selected modules: EMT, IFN signaling, and heme metabolism. Module analysis for selected cell types is shown. “*n*” represents the cell number in each cluster. Welch *t* test, *P* < 0.001.

Next, we performed module analysis with cancer-related gene sets, such as senescence, ROS production, IFN signaling, heme metabolism, and EMT genes. The module analysis depicted the alteration of cancer-related events by smoking in each cluster (Fig. 5A). Several modules were drastically altered between the smoker and never-smoker groups, such as IFN signaling in endothelial and myeloid clusters; EMT in epithelial, fibroblastic, and endothelial clusters; and mitophagy in lymphoid and myeloid clusters. Because increased expression of EMT module genes in endothelial clusters was observed, we examined the expression of endothelial-to-mesenchymal transition (EndMT) marker genes (*FN1*, *POSTN*, *VIM*; refs. 17, 46). These EndMT markers were significantly upregulated, suggesting that smoking induced EndMT in some endothelial clusters (Fig. 5B).

The module scores of ROS signaling significantly increased in the endothelial clusters. ROS signaling in endothelia induced inflammatory response and endothelial dysfunction (47, 48). In addition, myeloid cells enhanced IFN signaling in smoking lungs (Fig. 5B, bottom left). Autophagy in immune cells is important for cellular immunity, differentiation, and survival (49). Autophagy modules increased in immune cells and fibroblastic cells. Finally, increased senescence module scores were broadly observed across most cell types (Supplementary Fig. S12), suggesting that smoking induced aging in the lung. The module analysis of the cigarette-smoking lung atlas evidently indicated what cell types were influenced by smoking and how smoking affected these cells in the lung.

### Aging-related Gene Expression in the Integrated Atlas with Cigarette Smoking

**FIGURE 6:**
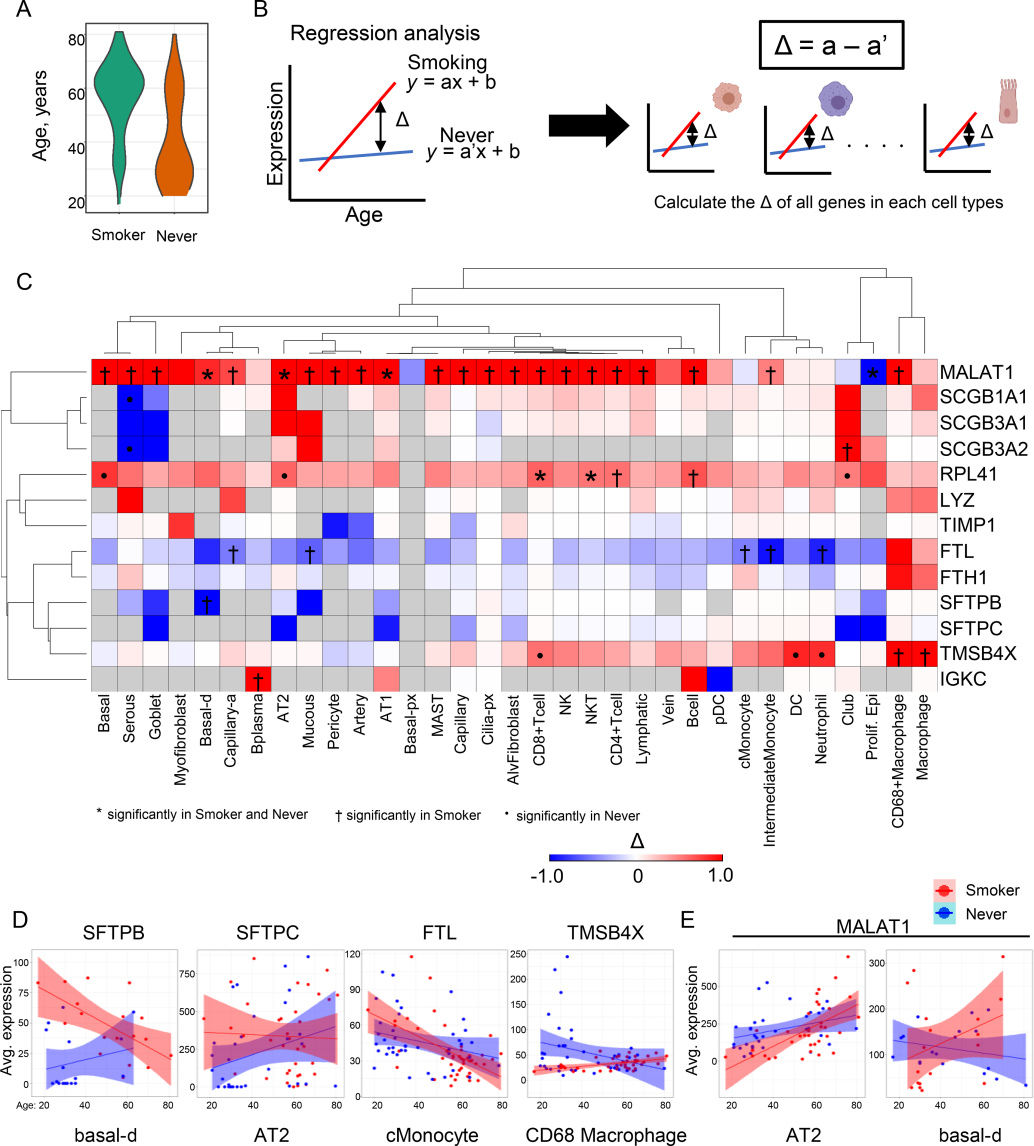
AGED analysis to identify genes related to advancing age and smoking. **A,** Age distributions of the smoker and never-smoker groups. **B,** Schematic of AGED analysis for determining gene alterations with advancing age in smokers. On the basis of regression analysis of single-cell transcriptome data with age, the differences in slopes (Δ) between the smoker and never-smoker groups in 39 cell clusters were calculated for all genes. The Δ values for selected genes were plotted in a heat map. **C,** Heat map of AGED analysis results. *P* value < 0.05: * significant in smokers and never-smokers, † significant in smokers, “•” significant in never-smokers. **D,** Representative correlation plots of *SFTPB* in basal-d cluster, *SFTPC* in AT2 cluster*, FTL* in cMonocyte cluster, and TMSB4X in CD68+Macrophage cluster. **E,** Representative plots of *MALAT1* expression in AT2 and basal-d clusters.

As the majority of the samples in the atlas had patient age information, we aimed to identify aging-related genes associated with cigarette smoking (Fig. 6A). We developed AGED analysis based on regression analysis with single-cell transcriptome data. Briefly, by using regression analysis with age and gene expression in the smoker and never-smoker groups, we calculated the differences in slopes (Δ) for all genes in 39 cell clusters (Fig. 6B; Supplementary Table S6). For selected genes that were obviously changed with advancing age between the smoker and never-smoker groups, the Δ values were plotted as AGED results in a heat map (Fig. 6C). These data showed that the lung surfactant proteins *SFTPC* and *SFTPB* decreased in several epithelial clusters with advancing age in the smoker (Fig. 6C and D, left). These lung surfactant proteins maintain the activation of alveolar macrophages and promote recovery from injuries induced by smoking (50). In addition, secretoglobins (*SCGB3A1*, *SCGB3A2*, and *SCGB1A1*) were also decreased in secretory goblet cells and serous cells with advancing age in the smokers. *MALAT1* is a well-known long noncoding RNA in lung cancer, and its expression contributes to malignancy (51, 52). AGED analysis showed that *MALAT1* expression increased in most cell types with advancing age in smokers (Fig. 6C and E), suggesting that the oncogenic risk associated with *MALAT1* increased with age. From the module analysis, heme metabolism was dysregulated in the myeloid cells of smoker lung (Fig. 5A). High expression of *TMSB4X* is contributed to poor prognosis and predicts the metastasis in lung carcinoma (52). *TMSB4X* increased in most cell types of immune clusters in the smoker lung (Fig. 6D, right). Finally, the expression level of *FTL* was significantly altered with advancing age in the smokers (Fig. 6C). In the cMonocyte clusters, *FTL* significantly decreased with smoking and aging. Collectively, the AGED analysis revealed changes in aging-related gene expression with smoking in each cell cluster.

## Discussion

In this study, we presented a human cigarette-smoking lung atlas, generated via the meta-analysis of 104 samples from eight public scRNA-seq datasets. Our integrated smoking atlas confirmed the alteration of gene expression in the lung at single-cell resolution and identified the early oncogenic events induced by cigarette smoking. In addition, the novel VARIED and AGED analyses revealed cell type and gene expressional diversity with smoking and age.

One of the significant contributions of this study is that the scMeta-analysis of integrated atlas identified expressional diversity in the early phase of lung squamous carcinoma at the single-cell level. In fact, expression analysis following VARIED revealed early oncogenic signaling in epithelial cells, expression changes in GWAS-related genes, and gender-dependent alterations in the smoking lung. DEGs analysis of basal-d clusters indicated that survival AKT-mTOR signaling, mitochondrial dysregulation, and sirtuin signaling pathways were altered in bronchial basal-d cells by smoking (Fig. 4B; Supplementary Table S5). In previous studies of the effects of smoking, genetic mutations in oncogenes and tumor suppressor genes were discovered (53–55). Bronchial epithelial cells from smokers have mutations in *TP53*, *NOTCH1*, *FAT1*, *CHEK2*, *PTEN*, *ARID1A,* and other genes (53). Mutations in *PTEN* contribute to the activation of AKT-mTOR signaling (56). *FAT1* controls mitochondrial functions (57), and its mutations induce the dysregulation of mitochondria. In addition, cigarette smoking promotes lung carcinogenesis by IKKβ- and JNK-dependent inflammation (58). DEGs analysis of basal-d clusters indicated that *ATF3*, *JUN,* and *FOS* expression levels were increased in the smoker basal-d cluster (Supplementary Table S3). High expression of *ATF3* expression contributes to tumor malignancy in lung cancer (59). Enrichment network analysis of basal-d DEGs showed that angiogenesis signaling, such as *VEGFA* and *FGF2*, is upregulated by smoking. Angiogenesis is an important event for tumor growth and remodeling of tumor microenvironment (60, 61). Our results suggested that smoking induces the remodeling of tumor microenvironment in the early phase of oncogenesis. Our module analysis showed enhancement of inflammatory signaling in the myeloid, fibroblastic, endothelia, and epithelial clusters. The integrated atlas confirmed the signaling related to genetic mutations induced by smoking.

AGED analysis showed that *MALAT1* expression increased in most cell types with advancing age in smokers. In the epithelial cells, the oncogenic function of MALAT1 is well known (51, 62); however, the function in stromal cells and immune cells remains unclear. Our results suggested that the oncogenic risk associated with MALAT1 is not only in the epithelial cells but also in the immune cells and stromal cells.

The first scMeta-analysis was performed to investigate severe acute respiratory syndrome coronavirus 2 (SARS-CoV-2)-related genes by The Human Cell Atlas Lung Biological Network (14). Furthermore, scMeta-analyses were reported for endothelial cells in the human and mouse lung (15) and liver-specific immune cells (16), which revealed the alteration of cell populations and expressional heterogeneity with single-cell resolution. In addition, the study of pan-cancer scRNA-seq cohorts revealed heterogeneity in tumor-infiltrating myeloid cell composition and the functions of cancer-specific myeloid cells (18). scMeta-analysis is a powerful tool and strategy to overcome the problem of sample bias in small clinical cohorts. In addition, our integrated atlas enabled us to perform single-cell analysis linked with clinical information in meta-cohorts such as AGED analysis, which identified aging-related gene expression with single-cell resolution. Furthermore, it revealed correlations in the alterations of gene expression associated with smoking and aging. Furthermore, scMeta-analyses incorporating additional clinical information will be helpful for understanding homeostasis and diseases.

Our study has limitations. First, differences in the tissue sampling and single-cell isolation methods generated bias in the cell populations used in this study. Generally, epithelial cells and immune cells tend to dominate in the scRNA-seq study. This bias could not be completely removed by computational normalization. In fact, our integrated atlas showed the differences in cell subpopulations in each dataset (Supplementary Fig. S2B). Therefore, this bias has the potential to influence the scRNA-seq methods, VARIED analysis and AGED analysis. As our results showed in Supplementary Fig. S10, VARIED analysis needs the large number of single cells in each cluster. In addition, there is a poor study for the molecular biology of basal-d cells in the lung. The omics data for bulk study and purification method of basal-d cells are not established. We consider that the further evaluation is needed using other scRNA-seq meta-dataset and bulk study. Next, clinical information such as smoking status, gender, and age depended on the collection in the primary studies. The atlas has only a simple classification: smoker or never-smoker; we could not consider detailed smoking information such as the amount of smoking, years of smoking, and Brinkman index (Supplementary Tables S1 and S2). In addition, patient age was significantly different between the smoker and never-smoker populations (Fig. 6A). Moreover, clinical information such as age and gender were not available for all datasets. In the future, it will be necessary to expand the integrated atlas following the publication of new appropriate datasets for a more robust analysis.

The integrated atlas presented herein contributed to the characterization of the alterations caused by cigarette smoking that are related to carcinogenesis of LUSC. However, lung cancer also develops in never-smokers, in whom lung adenocarcinoma is predominant (5, 6). scMeta-analysis focused on lung adenocarcinoma in different clinical status has the potential to reveal the nature of genetic carcinogenesis. As a future study, the integration of scRNA-seq data from normal lungs (never-smokers) and lung adenocarcinoma could be a feasible approach to discover the mechanism of carcinogenesis and elucidate the cellular diversity in lung adenocarcinoma. In addition, clinical scRNA-seq and scMeta-analysis will be powerful tools in combination with data from pan-cancer multiomics analyses, such as those in TCGA (33, 63, 64). Therefore, the integration of scMeta-analysis data with clinical and omics data paves the way for an in-depth understanding of the nature of cancer.

## Supplementary Material

Supplementary Figure S1 Establishment of the integrated lung scRNA-seq atlas with cigarette smoking status.

Supplementary Figure S2 Detailed information of the integrated lung scRNA-seq atlas.

Supplementary Figure S3 UMAP plots for selected marker genes.

Supplementary Figure S4 Epithelial cell analysis of smoker and never-smoker lungs.

Supplementary Figure S5 Fibroblast analysis of smoker and never-smoker lungs.

Supplementary Figure S6 Endothelial cell analysis of smoker and never-smoker lungs.

Supplementary Figure S7 Lymphoid cell analysis of smoker and never-smoker lungs.

Supplementary Figure S8 Myeloid cell analysis of smoker and never-smoker lungs.

Supplementary Figure S9 Cell cycle assessment across cell types in the cigarette smoking lung atlas.

Supplementary Figure S10 Verifying VARIED analysis by repeating random sampling.

Supplementary Figure S11 Analysis of GWAS-based squamous cell carcinoma-related genes.

Supplementary Figure S12 AGED analysis for cellular senescence.

Supplementary Table S1 A list of publicly-available 8 datasets for the atlas.

Supplementary Table S2 The details of integrated scRNA-seq samples in the atlas.

Supplementary Table S3 The DEGs list in basal-d clusters.

Supplementary Table S4 Signature genes of smoking basal-d clusters from DEGs analysis.

Supplementary Table S5 IPA canonical pathways in smoker basal-d cluster.

Supplementary Table S6 AGED analysis in all cell clusters.
