# Supplementary material for: Cancer-prone Phenotypes and Gene Expression Heterogeneity at Single-cell Resolution in Cigarette-smoking Lungs
Source: Cancer Res Commun. 2023 Nov 10;3(11):2280–91. doi: 10.1158/2767-9764.CRC-23-0195 (PMC10637260; doi:10.1158/2767-9764.CRC-23-0195)
Supplement: Supplementary Figure S1 — Establishment of the integrated lung scRNA-seq atlas with cigarette smoking status. [file crc-23-0195-s01.pdf]

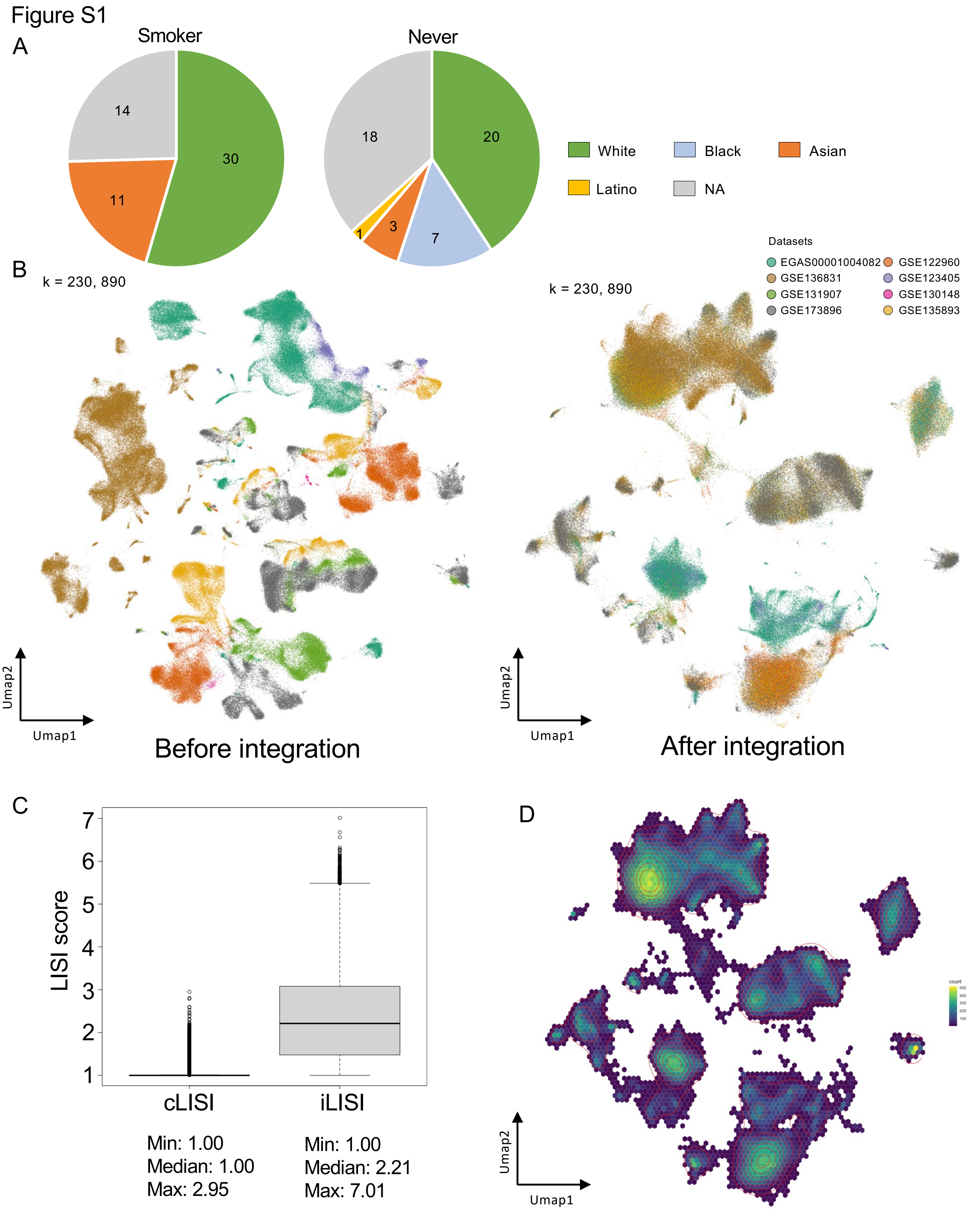

Supplementary Figure S1. Establishment of the integrated lung scRNA-seq atlas with cigarette smoking status.

A. The racial distributions of the smoker and never-smoker groups. B. A UMAP plots of before harmony integration and after harmony integration of 8 publicly datasets. C. cLISI score and iLISI score of the integrated atlas. D. A density UMAP plot of the integrated lung atlas.
