# Supplementary material for: Cancer-prone Phenotypes and Gene Expression Heterogeneity at Single-cell Resolution in Cigarette-smoking Lungs
Source: Cancer Res Commun. 2023 Nov 10;3(11):2280–91. doi: 10.1158/2767-9764.CRC-23-0195 (PMC10637260; doi:10.1158/2767-9764.CRC-23-0195)
Supplement: Supplementary Figure S2 — Detailed information of the integrated lung scRNA-seq atlas. [file crc-23-0195-s02.pdf]

Figure S2

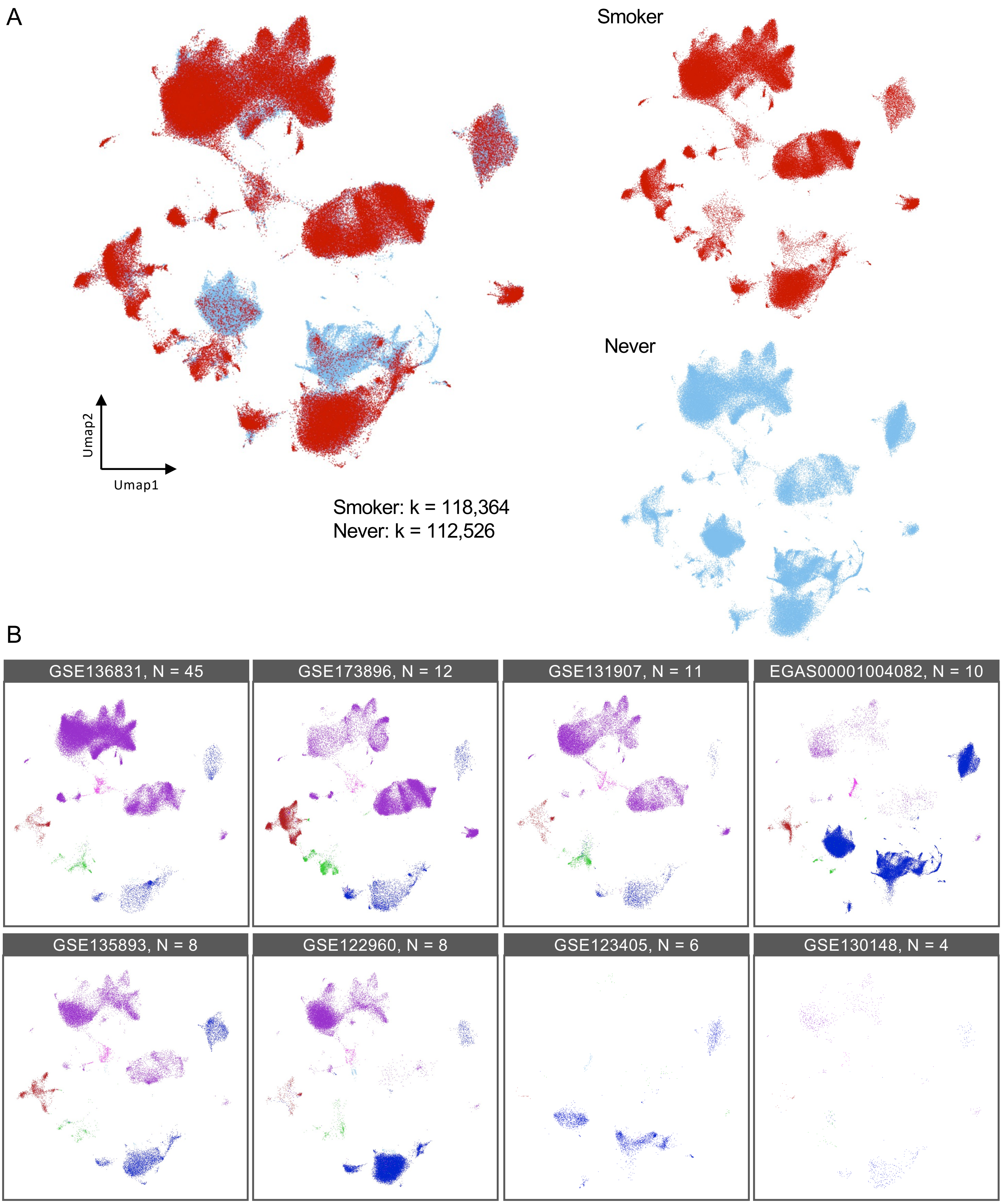

Supplementary Figure S2. Detailed information of the integrated lung scRNA-seq atlas.
A. UMAP plot of the integrated lung atlas with smoker/never-smoker information. B. Individual UMAP plots for each of 8 publicly available datasets. Blue: epithelia, purple: immune cells, red: endothelia, green: fibroblasts, pink: proliferating immune cells, and light blue: proliferating epithelia.
