# Supplementary material for: Cancer-prone Phenotypes and Gene Expression Heterogeneity at Single-cell Resolution in Cigarette-smoking Lungs
Source: Cancer Res Commun. 2023 Nov 10;3(11):2280–91. doi: 10.1158/2767-9764.CRC-23-0195 (PMC10637260; doi:10.1158/2767-9764.CRC-23-0195)
Supplement: Supplementary Figure S3 — UMAP plots for selected marker genes. [file crc-23-0195-s03.pdf]

Figure S3

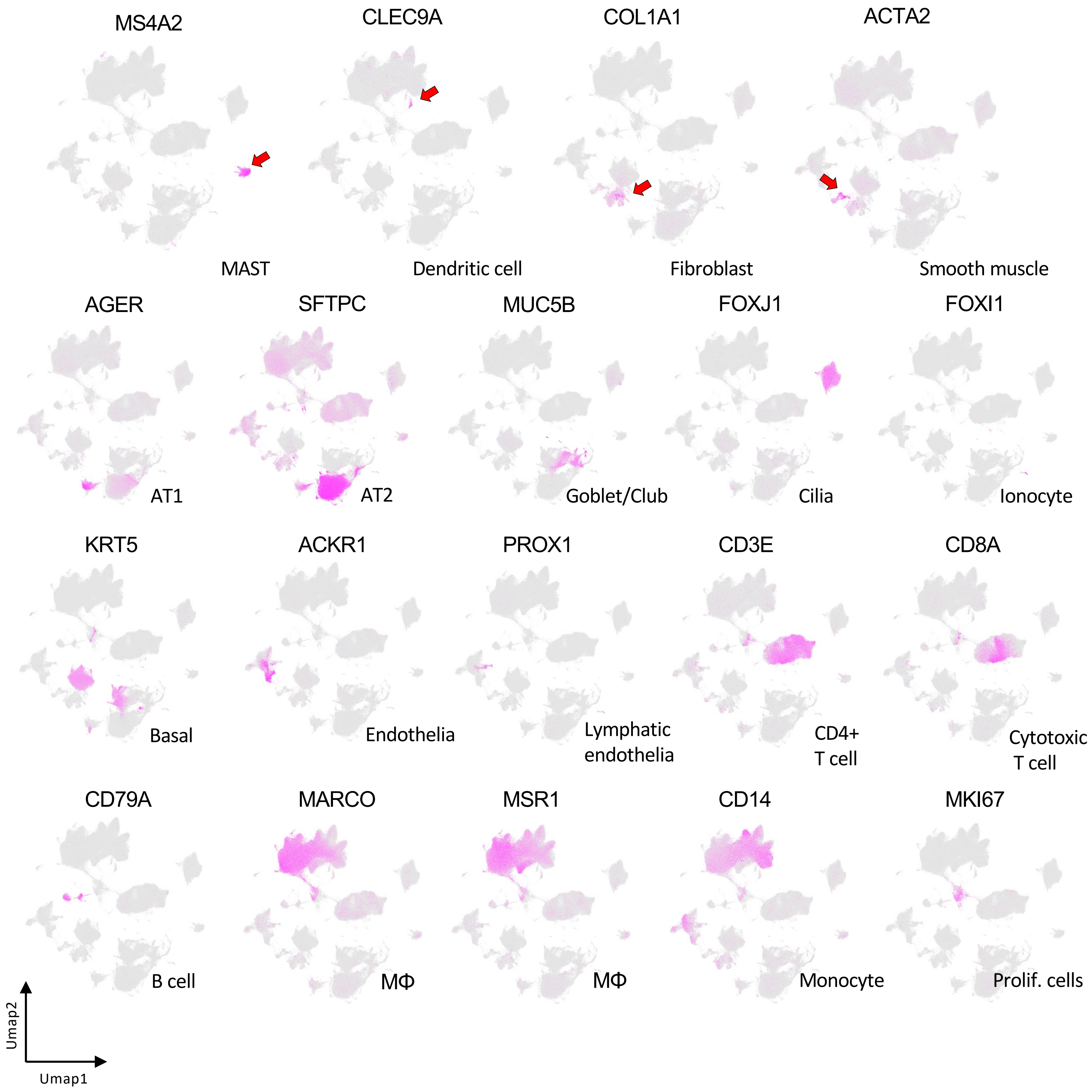

Supplementary Figure S3. UMAP plots for selected marker genes.
Feature plots of selected marker genes in all clusters. MS4A2 (MAST), CLEC9A (Dendritic cell), COL1A1 (Fibroblast), ACTA2 (Smooth muscle), AGER (AT1: Alveolar type1), SFTPC (AT2: Alveolar type2), MUC5B (Goblet / Club), FOXJ1 (Cilia), FOXI1 (Ionocyte), KRT5 (Basal), ACKR1 (Endothelia), PROX1 (Lymphatic endothelia), CD3E (CD4+ T cell), CD8A (Cytotoxic T cell), CD79A (B cell), MARCO (Macrophage), MSR1 (Macrophage), CD14 (Monocyte), and MKI67 (Proliferating cell) markers were shown as UMAP plot.
