# Supplementary material for: Cancer-prone Phenotypes and Gene Expression Heterogeneity at Single-cell Resolution in Cigarette-smoking Lungs
Source: Cancer Res Commun. 2023 Nov 10;3(11):2280–91. doi: 10.1158/2767-9764.CRC-23-0195 (PMC10637260; doi:10.1158/2767-9764.CRC-23-0195)
Supplement: Supplementary Figure S4 — Epithelial cell analysis of smoker and never-smoker lungs. [file crc-23-0195-s04.pdf]

Figure S4

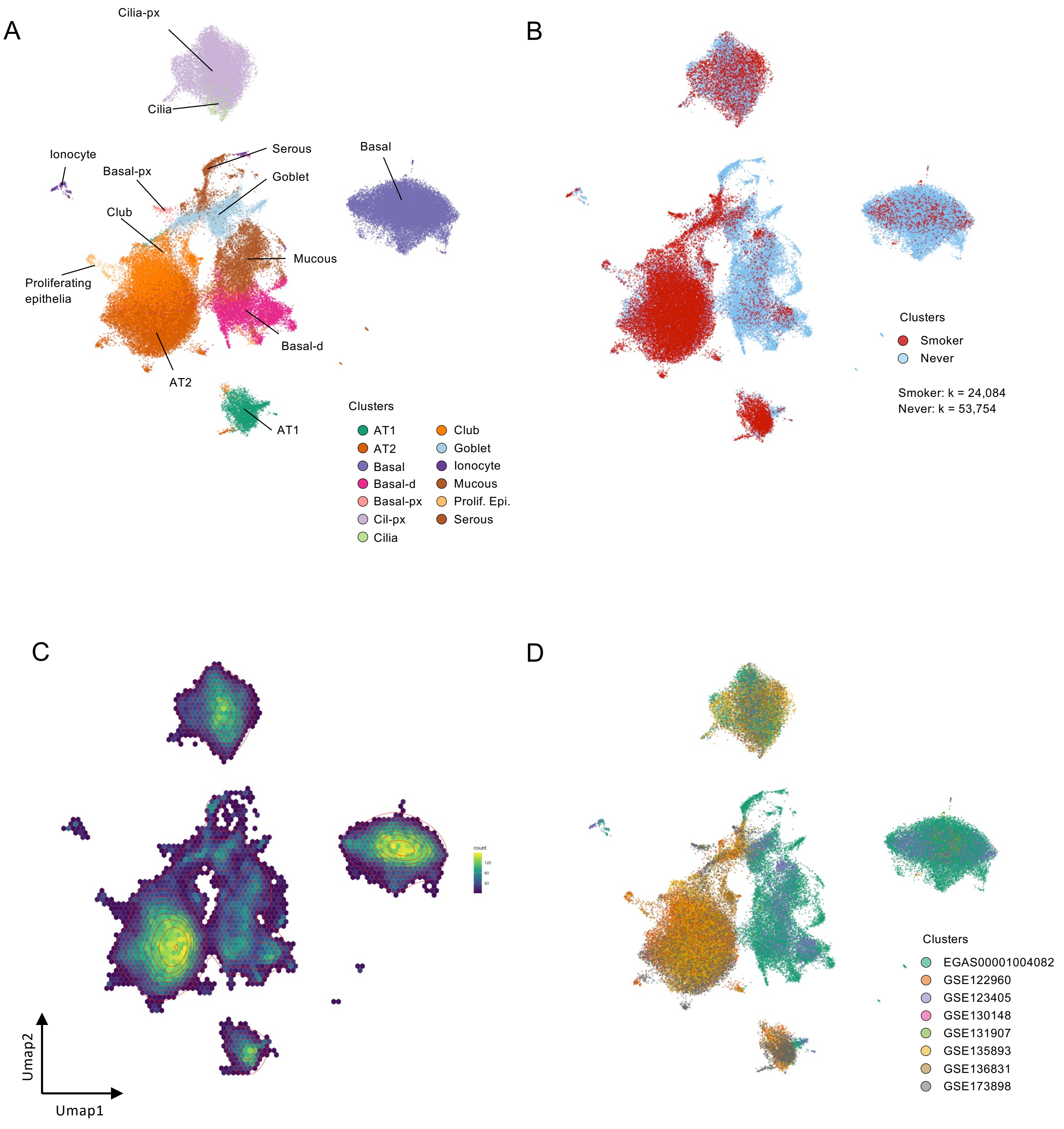

Supplementary Figure S4. Epithelial cell analysis of smoker and never-smoker lungs.
A. UMAP plot of 77,838 epithelial cells and proliferating epithelial cells from the UMAP shown in Figure 1D. The dots are labeled by cell type as identified by marker expression profiles. Twelve distinct clusters were identified. B. UMAP plot with sample status. Smoker: k = 24,084; never-smoker: k = 53,754. C. Density UMAP plot of epithelial cell clusters. D. UMAP plot of epithelial cell clusters marked by dataset.
