# Supplementary material for: Cancer-prone Phenotypes and Gene Expression Heterogeneity at Single-cell Resolution in Cigarette-smoking Lungs
Source: Cancer Res Commun. 2023 Nov 10;3(11):2280–91. doi: 10.1158/2767-9764.CRC-23-0195 (PMC10637260; doi:10.1158/2767-9764.CRC-23-0195)
Supplement: Supplementary Figure S5 — Fibroblast analysis of smoker and never-smoker lungs. [file crc-23-0195-s05.pdf]

Figure S5

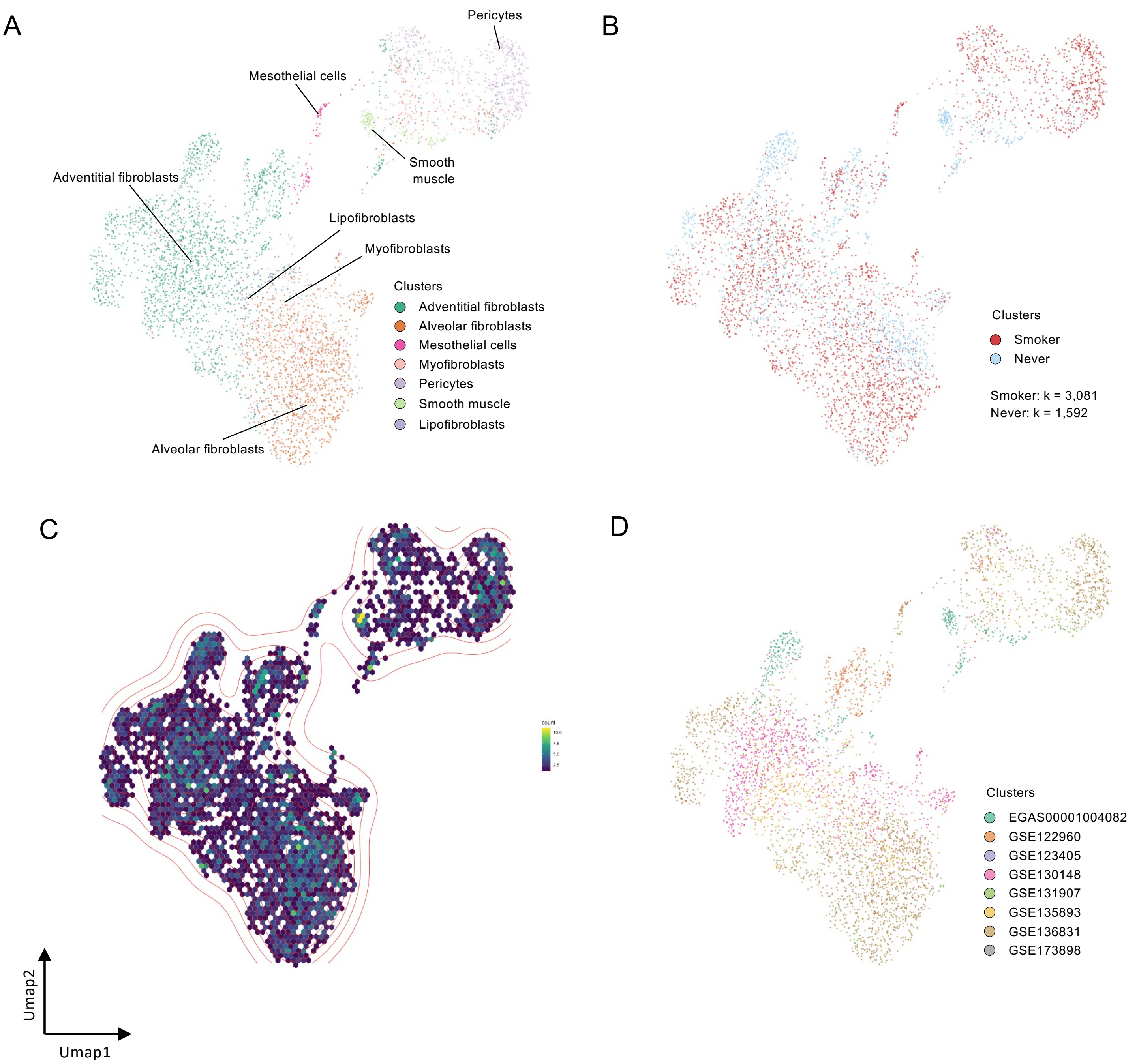

Supplementary Figure S5. Fibroblast analysis of smoker and never-smoker lungs.
A. UMAP plot of 4,673 fibroblasts from the UMAP shown in Figure 1B. The dots are labeled by cell type as identified by marker expression profiles. Seven distinct clusters were identified. B. UMAP plot with sample status. Smoker: k = 3,081; never-smoker: k = 1,592. C. Density UMAP plot of fibroblastic cell clusters. D. UMAP plot of fibroblastic cell clusters marked by dataset.
