# Supplementary material for: Cancer-prone Phenotypes and Gene Expression Heterogeneity at Single-cell Resolution in Cigarette-smoking Lungs
Source: Cancer Res Commun. 2023 Nov 10;3(11):2280–91. doi: 10.1158/2767-9764.CRC-23-0195 (PMC10637260; doi:10.1158/2767-9764.CRC-23-0195)
Supplement: Supplementary Figure S6 — Endothelial cell analysis of smoker and never-smoker lungs. [file crc-23-0195-s06.pdf]

Figure S6

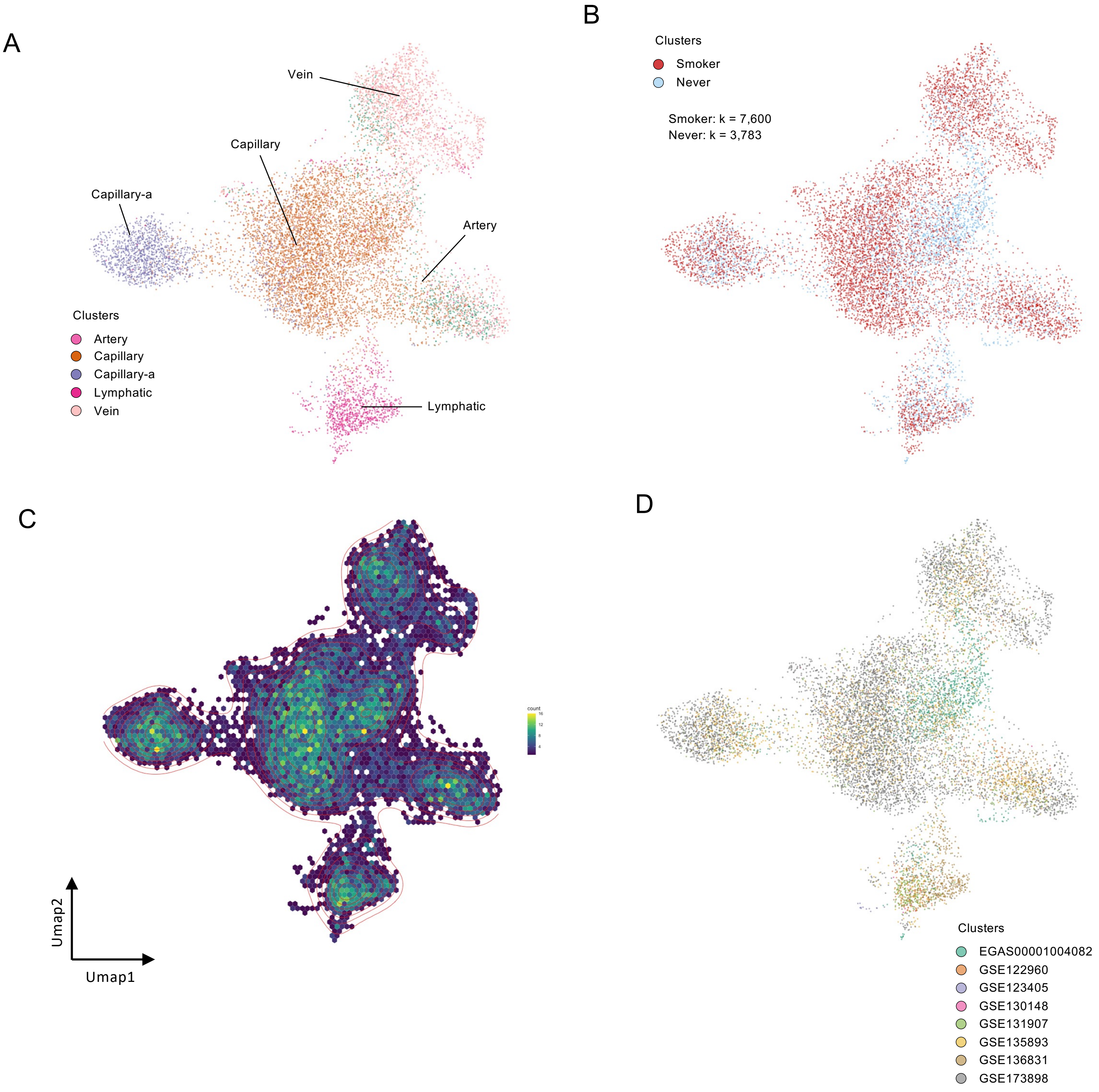

Supplementary Figure S6. Endothelial cell analysis of smoker and never-smoker lungs.
A. UMAP plot of 11,383 endothelial cells from the UMAP shown in Figure 1B. The dots are labeled by cell type as identified by marker expression profiles. Seven distinct clusters were identified. B. UMAP plot with sample status. Smoker: k = 7,600; never-smoker: k = 3,783. C. Density UMAP plot of endothelial cell clusters. D. UMAP plot of endothelial cell clusters marked by dataset.
