# Supplementary material for: Cancer-prone Phenotypes and Gene Expression Heterogeneity at Single-cell Resolution in Cigarette-smoking Lungs
Source: Cancer Res Commun. 2023 Nov 10;3(11):2280–91. doi: 10.1158/2767-9764.CRC-23-0195 (PMC10637260; doi:10.1158/2767-9764.CRC-23-0195)
Supplement: Supplementary Figure S8 — Myeloid cell analysis of smoker and never-smoker lungs. [file crc-23-0195-s08.pdf]

Figure S8

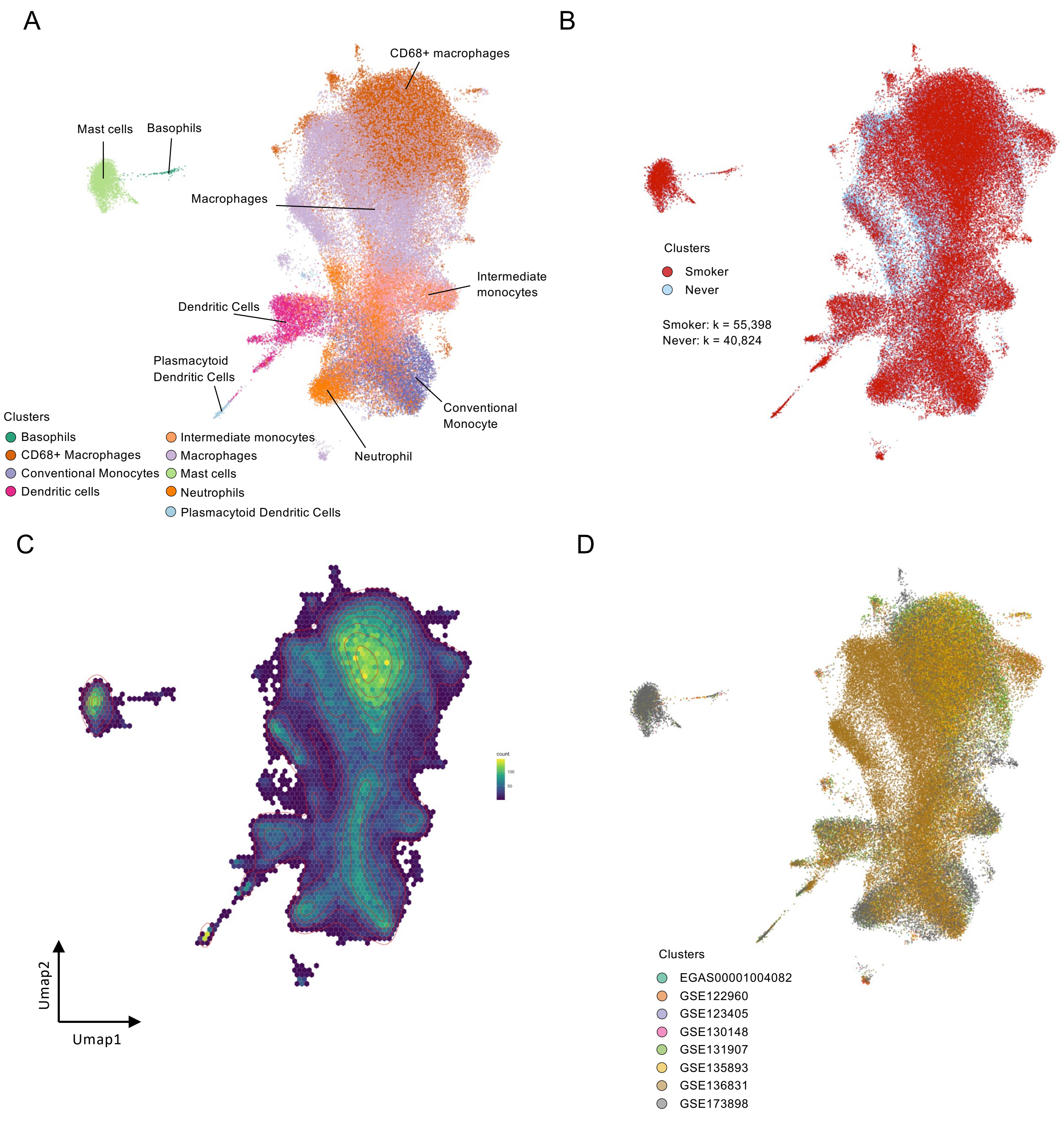

Supplementary Figure S8. Myeloid cell analysis of smoker and never-smoker lungs.
A. UMAP plot of 96,222 myeloid cells. The dots are labeled by cell type as identified by marker expression profiles. Eight distinct clusters were identified. B. UMAP plot with sample status. Smoker: k = 55,398; never-smoker: k = 40,824. C. Density UMAP plot of myeloid cell clusters. D. UMAP plot of myeloid cell clusters marked by dataset.
