# Supplementary material for: Cancer-prone Phenotypes and Gene Expression Heterogeneity at Single-cell Resolution in Cigarette-smoking Lungs
Source: Cancer Res Commun. 2023 Nov 10;3(11):2280–91. doi: 10.1158/2767-9764.CRC-23-0195 (PMC10637260; doi:10.1158/2767-9764.CRC-23-0195)
Supplement: Supplementary Figure S9 — Cell cycle assessment across cell types in the cigarette smoking lung atlas. [file crc-23-0195-s09.pdf]

Figure S9

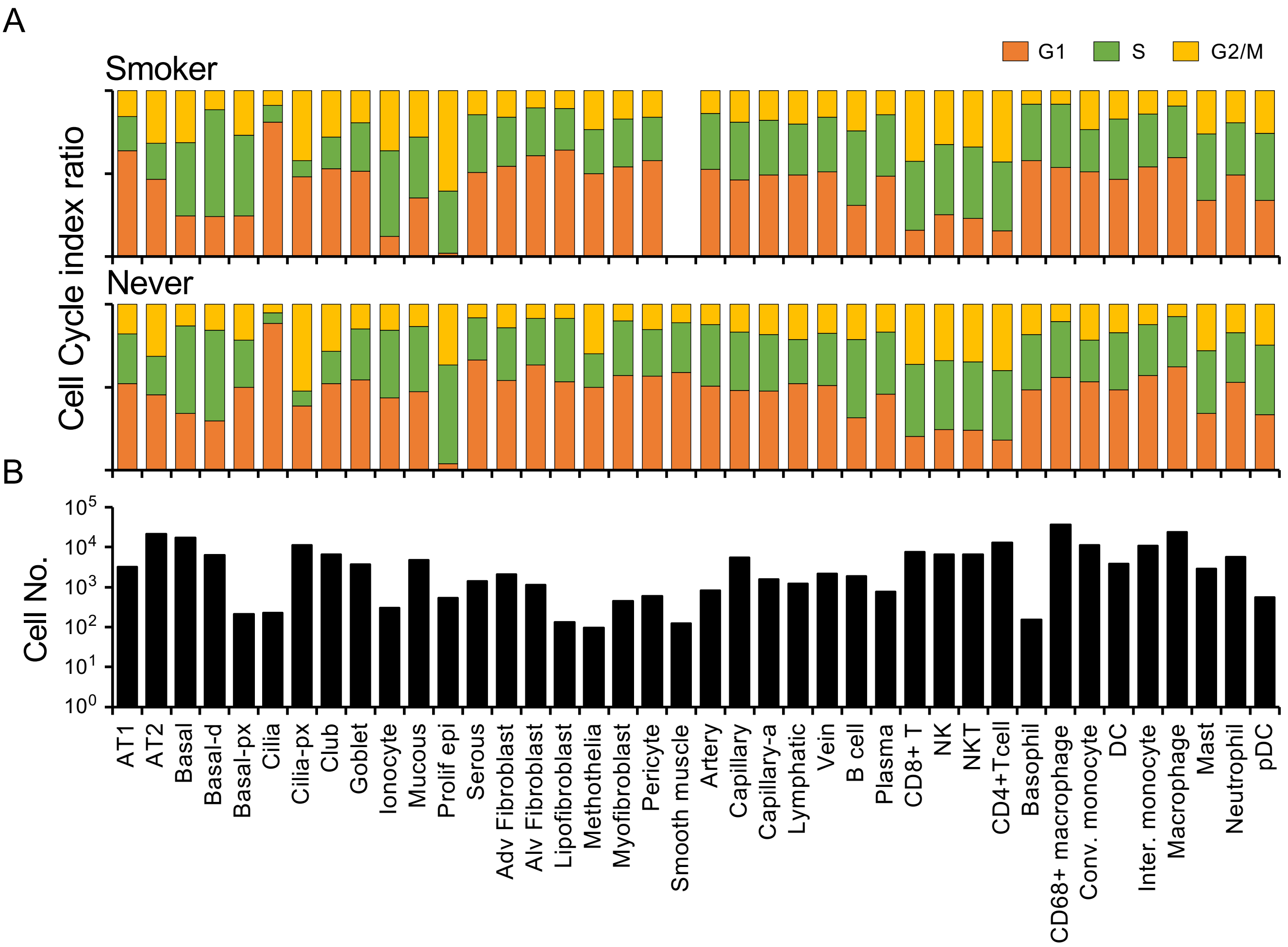

Supplementary Figure S9. Cell cycle assessment across cell types in the cigarette smoking lung atlas.

A. Cell cycle phase prediction based on scRNA-seq profiles. G1, S, and G2/M phases are predicted in each cell type. Top: smoker; bottom: never-smoker. B. Cell numbers across the cell types.
