# Supplementary material for: Cancer-prone Phenotypes and Gene Expression Heterogeneity at Single-cell Resolution in Cigarette-smoking Lungs
Source: Cancer Res Commun. 2023 Nov 10;3(11):2280–91. doi: 10.1158/2767-9764.CRC-23-0195 (PMC10637260; doi:10.1158/2767-9764.CRC-23-0195)
Supplement: Supplementary Figure S10 — Verifying VARIED analysis by repeating random sampling. [file crc-23-0195-s10.pdf]

Figure S10

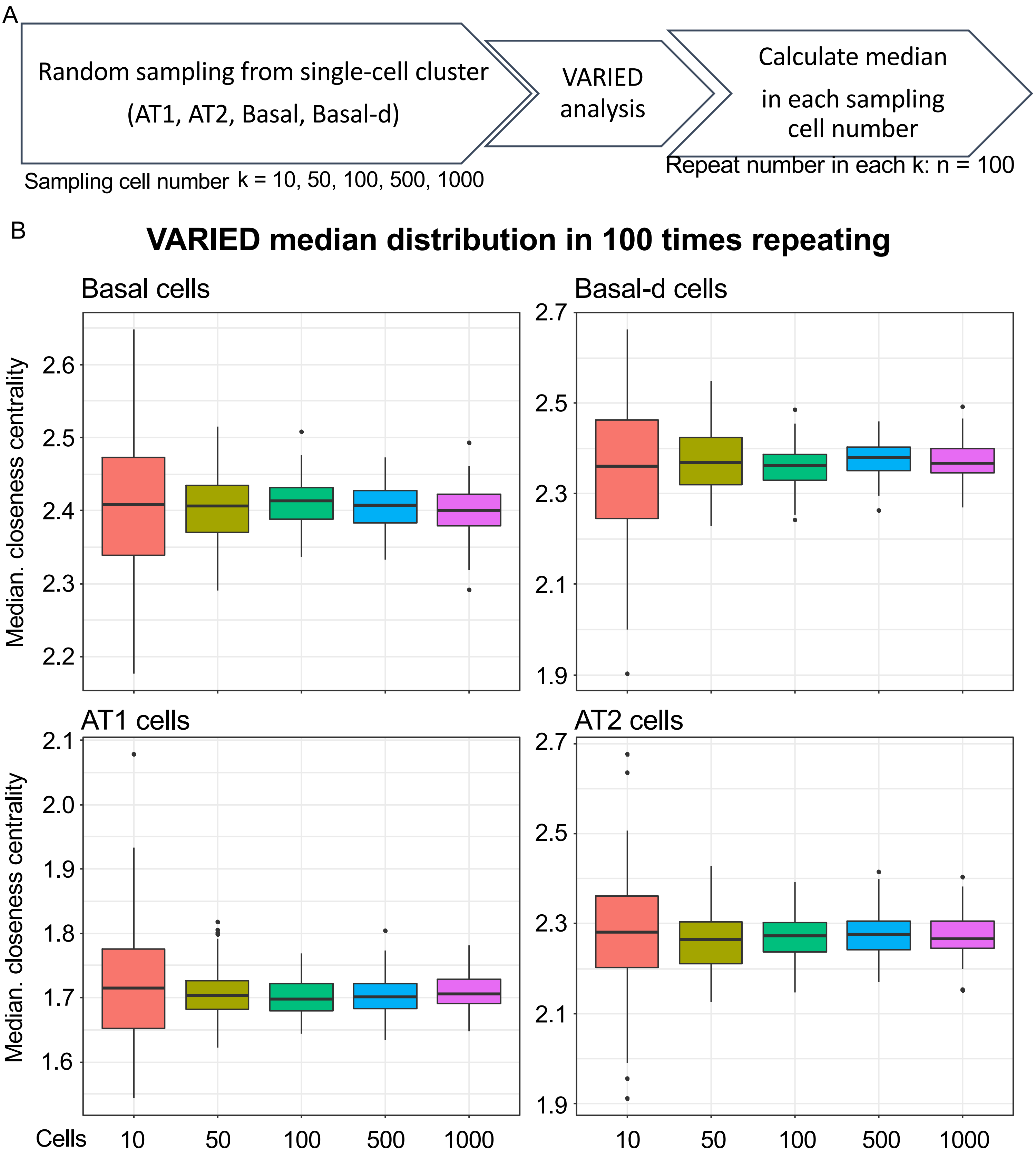


**Supplementary Figure S10. Verifying VARIED analysis by repeating random sampling.**
A. Overview of the accuracy test of VARIED analysis. In first step, the random sampling of cells from the clusters (cell number: k = 10, 50, 100, 500, and 1000) were performed in R. In second step, sampled cells were transformed to correlational network, and performed VARIED analysis. In last step, the median of VARIED score in random sampled cells. It returned to first step. Median calculation was repeated 100 times. B. The boxplot showed the distribution of VARIED score median in repeating random sampling test with each sampling cell number.
