# Supplementary material for: Cancer-prone Phenotypes and Gene Expression Heterogeneity at Single-cell Resolution in Cigarette-smoking Lungs
Source: Cancer Res Commun. 2023 Nov 10;3(11):2280–91. doi: 10.1158/2767-9764.CRC-23-0195 (PMC10637260; doi:10.1158/2767-9764.CRC-23-0195)
Supplement: Supplementary Figure S11 — Analysis of GWAS-based squamous cell carcinoma-related genes. [file crc-23-0195-s11.pdf]

Figure S11

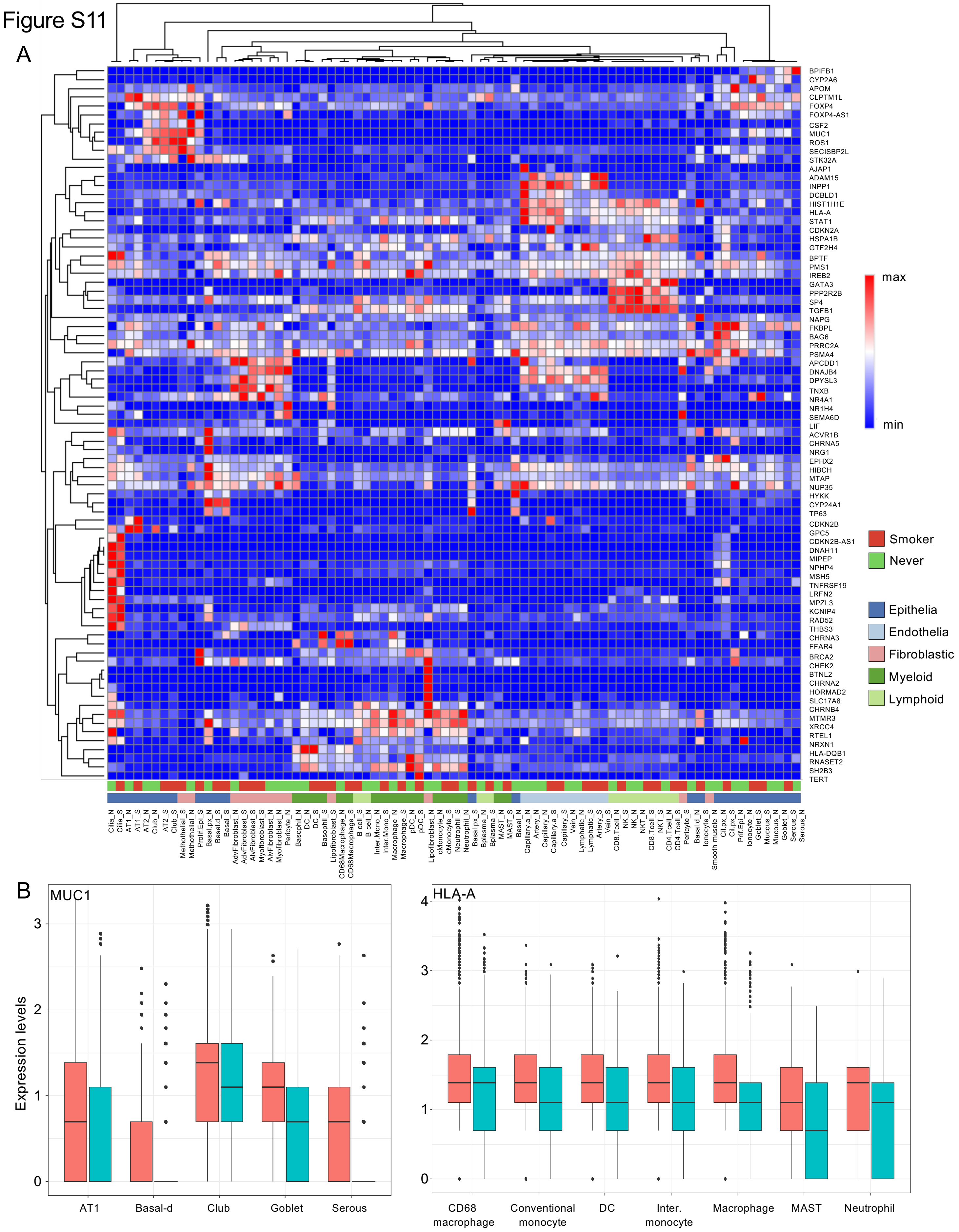

Supplementary Figure S11. Analysis of GWAS-based squamous cell carcinoma-related genes.
A. Expression profiles of 92 lung squamous cell carcinoma GWAS genes in all cell types based on the cigarette smoking lung atlas. B. MUC1 expression in selected epithelial clusters between the smoker and never-smoker groups. HLA-A expression in selected myeloid clusters between the smoker and never-smoker groups. Welch's t test.
