# Supplementary material for: Cancer-prone Phenotypes and Gene Expression Heterogeneity at Single-cell Resolution in Cigarette-smoking Lungs
Source: Cancer Res Commun. 2023 Nov 10;3(11):2280–91. doi: 10.1158/2767-9764.CRC-23-0195 (PMC10637260; doi:10.1158/2767-9764.CRC-23-0195)
Supplement: Supplementary Figure S12 — AGED analysis for cellular senescence. [file crc-23-0195-s12.pdf]

Figure S12

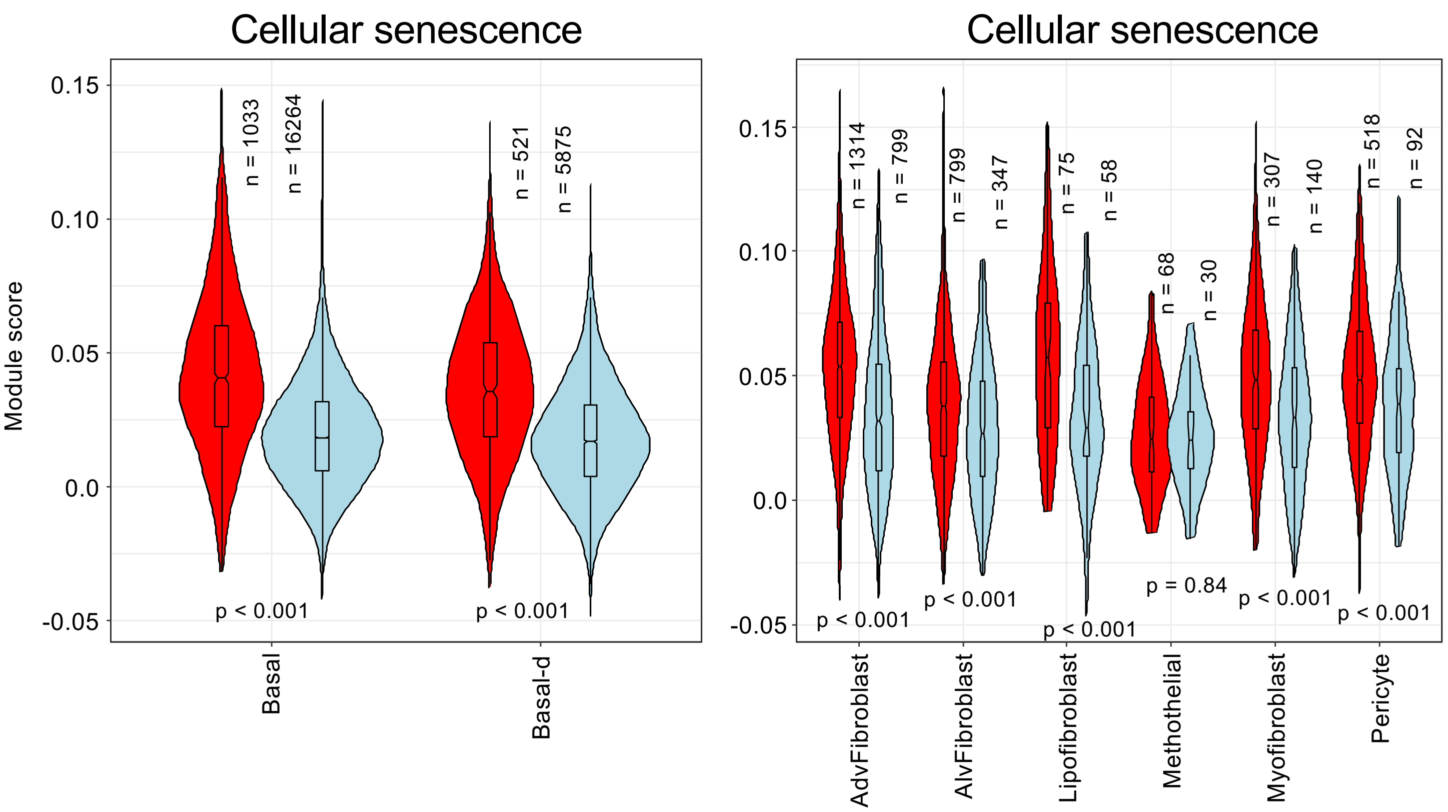

Supplementary Figure S12. AGED analysis for cellular senescence.
A heatmap of AGED analysis results for cellular senescence in basal, basal-d, AdvFibroblast, AlvFibroblast, Lipofibroblast, methothelial, myofibroblast, and pericyte between smokers and never-smokers. “n” represents the cell number in each cluster. Welch’s t test.
